# Galectin-3 Regulates γ-Herpesvirus Specific CD8 T Cell Immunity

**DOI:** 10.1016/j.isci.2018.10.013

**Published:** 2018-10-17

**Authors:** Manpreet Kaur, Dhaneshwar Kumar, Vincent Butty, Sudhakar Singh, Alexandre Esteban, Gerald R. Fink, Hidde L. Ploegh, Sharvan Sehrawat

**Affiliations:** 1 Indian Institute of Science Education and Research Mohali, Sector 81 SAS Nagar, PO Manauli, Mohali, Knowledge City 140306, Punjab, India; 2 Whitehead Institute for Biomedical Research, 9 Cambridge Center, Cambridge 02142 MA, USA

**Keywords:** Immune Response, Immunology, Transcriptomics, Virology

## Abstract

To gain insights into the molecular mechanisms and pathways involved in the activation of γ-herpesvirus (MHV68)-specific T cell receptor transnuclear (TN) CD8^+^ T cells, we performed a comprehensive transcriptomic analysis. Upon viral infection, we observed differential expression of several thousand transcripts encompassing various networks and pathways in activated TN cells compared with their naive counterparts. Activated cells highly upregulated galectin-3. We therefore explored the role of galectin-3 in influencing anti-MHV68 immunity. Galectin-3 was recruited at the immunological synapse during activation of CD8^+^ T cells and helped constrain their activation. The localization of galectin-3 to immune synapse was evident during the activation of both naive and memory CD8^+^ T cells. Galectin-3 knockout mice mounted a stronger MHV68-specific CD8^+^ T cell response to the majority of viral epitopes and led to better viral control. Targeting intracellular galectin-3 in CD8^+^ T cells may therefore serve to enhance response to efficiently control infections.

## Highlights

• A comprehensive transcriptomic analysis on γ-HV-expanded TN cells was performed
• CD8 T cells upregulated Gal-3 that migrated intracellularly toward immune synapse
• Stimulated naive and memory cells displayed galectin-3 recruitment to immune synapse
• γ−HV-infected Gal-3 KO mice mounted better CD8^+^ T cells and controlled virus better

## Graphical Abstract

**Figure:**
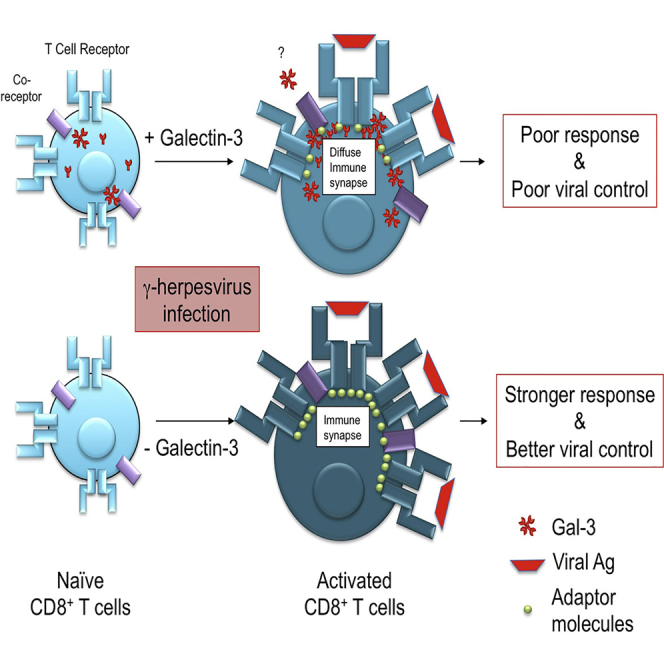

## Introduction

Timely induction of an adaptive immune response and the formation of effective immunological memory are essential for protective immunity against infectious diseases (Ahmed and Gray, 1996). Appropriately activated CD8^+^ T cells help control intracellular pathogens through recognition of peptides derived from pathogens in the context of class I major histocompatibility complex (MHC) products. Induction of a CD8^+^ T cell response requires the processing of three types of signal, delivered via peptide-MHC (p-MHC) complexes, co-stimulatory molecules, and the prevailing cytokine milieu. Activated CD8^+^ T cells expand to become effector cells that lyse their targets and so eliminate the intracellular pathogen (Zinkernagel, 1996). Several mechanisms then engage to regulate this response to limit possible damage caused by hyperactive immune cells (Ahmed and Gray, 1996).

Most pathogens establish an intricate relationship, manifested at many stages, with their host to ensure transmission, including invasion and the establishment of a productive infection. Barring a few exceptions, pathogen-specific CD8^+^ T cell responses are usually polyclonal in nature and recognize multiple epitopes specified by the invading pathogen (Wong and Pamer, 2003). This applies in particular to complex pathogens such as poxviruses and herpesviruses (HVs) in their natural hosts (Amanna et al., 2006, Freeman et al., 2010, Gredmark-Russ et al., 2008, Moutaftsi et al., 2006). γ-HVs are species-specific pathogens, and therefore analysis of anti-γ-HV responses requires a natural host. Infection of mice with murine herpesvirus 68 (MHV68) is one of the most accessible model systems to study anti-γ-HV immunity and immunopathology (Nash and Dutia, 2008, Nash et al., 2001, Sehrawat et al., 2018). Various immune mediators induced in MHV68-infected mice display similarity to those induced during γ-HV infections in humans (Barton et al., 2011). Previously, we generated MHV68-specific CD8^+^ T cell receptor transnuclear (TCR TN) mice by somatic cell nuclear transfer approach to investigate the contribution of CD8^+^ T cells to viral control (Sehrawat et al., 2012). TCR TN mice use the physiological rearrangements of the endogenous antigen receptor loci. Therefore, CD8^+^ TCR TN mice are likely to yield physiologically relevant primary T cell populations to investigate their responsiveness during infection (Kirak et al., 2010, Sehrawat et al., 2012).

The efficiency with which T cells engage antigen-presenting cells (APCs) in an immunological synapse regulates their activation, differentiation, and functionality (Dustin and Cooper, 2000, Fooksman et al., 2010, Mempel et al., 2004, Viola et al., 1996). The components of the immune synapse, such as the TCR, co-receptors (CD3 with all its subunits), lineage differentiation surface glycoproteins such as CD4 and CD8, adhesion molecules, and phosphatases (CD45), are extensively decorated with carbohydrates (Qian and Weiss, 1997). Therefore carbohydrate-binding proteins (lectins) such as members of the galectin family may be critical in the formation, stabilization, and disassembly of the immunological synapse. Galectins may also affect the differentiation of T cells (Hsu et al., 2009). Galectins bind to a variety of glycosylated proteins, expressed intracellularly or on the cell surface (van Kooyk and Rabinovich, 2008). Such interactions are either mediated by galectin-glycan lattices or through specific receptor-ligand pairs (van Kooyk and Rabinovich, 2008). At least 15 different galectins have been identified, and all have at least one conserved carbohydrate recognition domain (CRD), consisting of approximately 130 amino acids (Rabinovich et al., 2002). Although galectins do not have classical secretory signals, some can nonetheless be released into the extracellular space (Hughes, 1999).

We performed RNA sequencing (RNA-seq) on MHV68-specific naive and activated CD8^+^ TCR TN T cells to gain insight into their function and phenotype. Activated TN cells isolated from virus-infected mice differentially expressed several thousand transcripts. We discovered several novel transcripts whose function remains to be fully defined in T cell biology. Among these, we found strong upregulation of galectin-3 in the virus-specific CD8^+^ T cells activated and expanded in response to the MHV68 infection. We, therefore, investigated the role of galectin-3 in the activation of CD8^+^ T cells during MHV68 infection, as its contribution in anti-viral CD8^+^ T cell immunity remains ill defined. Some studies have suggested a regulatory role of galectin-3 in CD8^+^ T cell responses in autoimmune diseases and tumors (Gordon-Alonso et al., 2017, Kouo et al., 2015). In the tumor microenvironment, the extracellular galectin-3 interacted with effector molecule, interferon (IFN)-γ, owing to its extensive glycosylation and dampened its protective function against the developing tumor (Gordon-Alonso et al., 2017). We demonstrate that galectin-3 is recruited at the immunological synapse but predominantly acts intracellularly within CD8^+^ T cells engaged to cognate peptide displayed by MHC I both during the primary and memory response. CD8^+^ T cells lacking galectin-3 expanded more vigorously and produced enhanced cytokines when compared with wild-type (WT) CD8^+^ T cells. Furthermore, galectin-3 knockout (KO) mice mounted a stronger virus-specific CD8^+^ T cell response against most of the investigated epitopes and controlled virus better. Therefore, modulating the galectin-3 response pattern in CD8^+^ T cells may serve as a strategy to enhance their function.

## Results

### Phenotype of Naive and MHV68-Stimulated TCR TN CD8^+^ T Cells

**Figure 1:**
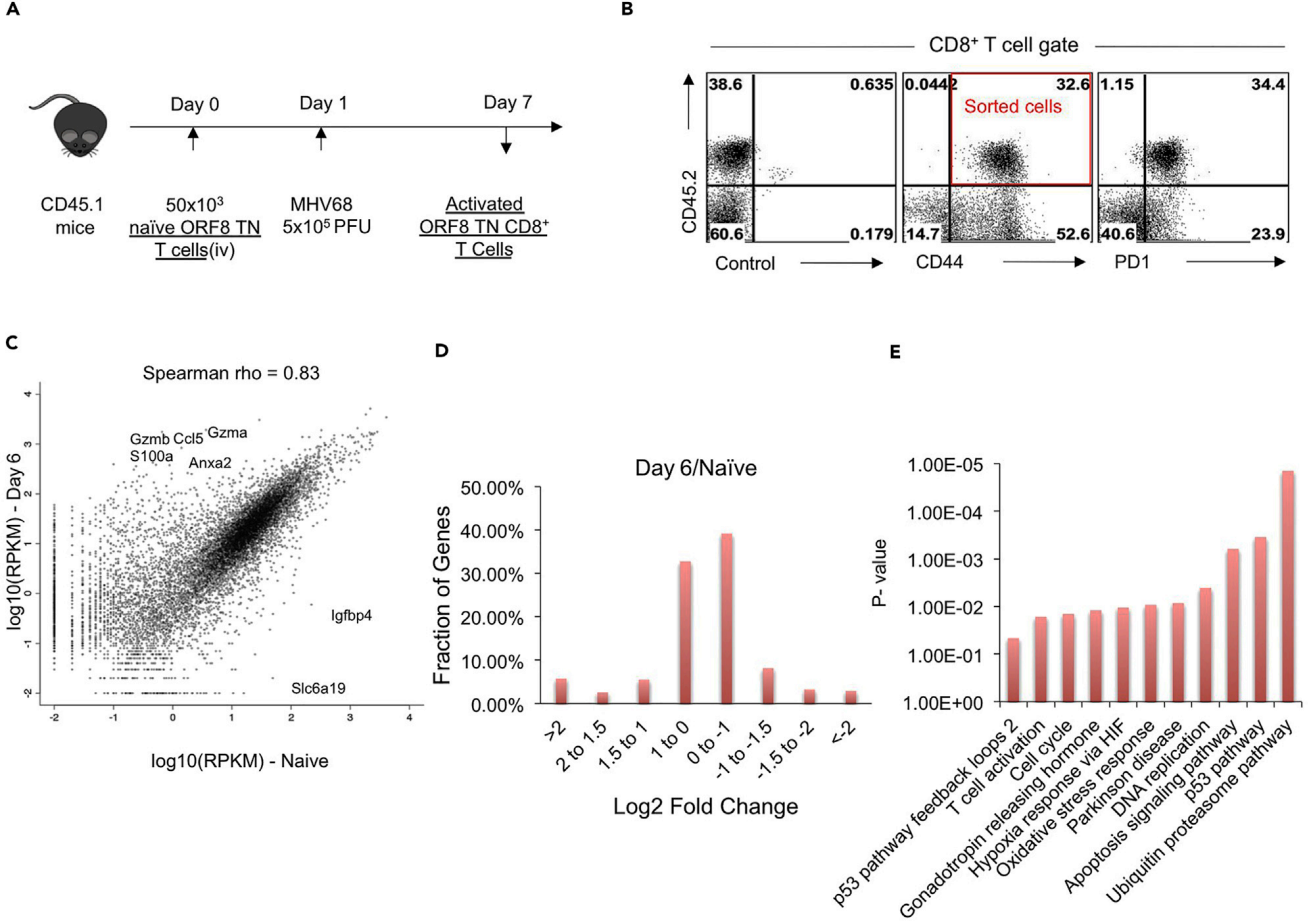
RNA-Seq Analysis of Naive and MHV68-Expanded ORF8 TCR TN CD8^+^ T Cells (A) Schematic of the experiment. 50 × 10^3^ Rag1^−/−^ K^b^-ORF8 TCR TN CD8^+^ T cells were adoptively transferred in CD45.1 congenic C57BL/6 mice, and recipients were infected with 5 × 10^5^ plaque-forming unit (PFU) of MHV68 i.p. On 6 dpi, spleens were isolated and single-cell suspensions were analyzed flow cytometrically using a panel of indicated cell surface markers. (B) Representative FACS plots showing the phenotypic markers expressed by CD45.2-positive cell (Rag1^−/−^ K^b^-ORF8 TCR TN CD8^+^ T cells) activated and expanded in response to viral infection. (C) Sorted CD45.2^+^ CD44^hi^ cells that were expanded due to MHV68 infection and their naive counterparts were used for RNA sequencing. Scatterplot shows the differential expression of transcripts in naive and 6 dpi activated TN CD8^+^ T cells. (D and E) (D) Bar diagram shows the extent by which the fraction of genes are differentially expressed. (E) Gene ontology panther pathway analysis of differentially expressed transcripts in ORF8 TCR TN CD8^+^ T cells for the biological processes represented.

We compared the transcriptome of activated MHV68-specific ORF8 TCR TN CD8^+^ T cells (Sehrawat et al., 2012) with their naive counterparts to gain insight into their function and phenotype. Both naive and activated CD8^+^ T cells were obtained from genetically comparable TCR TN mice (see schematic, Figure 1A). TN CD8^+^ T cells expanded massively in response to MHV68 infection in congenic (CD45.1) mice that received 50 × 10^3^ open reading frame (ORF) TN cells before infection. At 6 days post infection (dpi), 35%–40% of the total CD8^+^ T cells in spleens of infected mice were composed of donor ORF8 TCR TN cells. Cells that responded to viral infection had increased surface display of the activation markers such as CD44 and program death 1 (PD1) (Figure 1B), demonstrating a fresh recruitment of these cells in the course of the infection. Most of these cells produced IFN-γ in Intracellular cytokine staining (ICCS) assays (Sehrawat et al., 2012). We performed fluorescence-activated cell sorting (FACS) of activated ORF8 TCR TN CD8^+^ T cells for further analysis as shown in Figure 1B.

### RNA-Seq Data and Assessment of Its Quality

FACS-purified naive cells and activated cells were processed for RNA isolation. Libraries were constructed and subjected to paired-end sequencing. Approximately 1 million reads of excellent quality for each sample were obtained (Figures 1C and S1A). A high fraction of uniquely mapping reads having a minimal amount of ribosomal RNA sequences were obtained. Further analysis of RNA-seq data showed a high percentage of junction reads, good exon/intron as well as exon/intergenic ratios, and minimal 3'/5′ bias in sequence coverage (Figure S1A). The expression of some genes, as exemplified by β_2_m, class I MHC, and Tap1, did not change between naive and activated TN cells and served as a point of reference with which to compare the observed changes (Figure S1B). Normalized RPKM and a log_2_ fold change of different genes from naive and activated TN cells are shown in Table S1 and in a scatterplot (Figure 1C). Ensembl entries (n = 11,689) also present in our RNA-seq data with more than 5 reads in either samples are shown in a scatterplot (Figure 1C). The extent of differential expression of a large majority of genes in activated when compared with naive ORF8 TCR TN CD8^+^ T cells was up to less than 2-fold (Figure 1D). Top hits that were differentially expressed in TN cells are discussed in the following sections.

#### Analysis of Transcriptome of Naive and Activated TCR TN CD8^+^ T Cells

Our initial analyses involved the number of reads for the α (TRAV10N801, TRAJ33*01) and β chains (TRBV31*01, D1*01 and TRBJ2-2*01) of the specific TCR used by ORF8 TCR TN CD8^+^ T cells (Sehrawat et al., 2012). We found a high degree of sequence coverage across the expected TCR α and β chains (Figure S1C). As TCR expression is downregulated in stimulated T cells in the acute phase of response, fewer reads were obtained for the TCR chains in activated CD8^+^ T cells (Figure S1C).

Of the several thousand transcripts differentially expressed in activated TN CD8^+^ T cells, many have not been reported to exhibit a similar expression pattern Table S1. We also compared our RNA-seq data with the transcriptome of two of the commonly used CD8^+^ TCR transgenic (tg) mice (OT1 and P14) as these mice have provided excellent insights into the differentiation pathways of antigen-specific CD8^+^ T cells (Best et al., 2013, Wherry et al., 2007). The comparison revealed that more transcripts were differentially expressed in TCR TN CD8^+^ T cells (Figures S1D and S1E, Table S1). The varying TCR affinities of TN T cells when compared with the tg cells, the usage of endogenous TCR loci for receptor assembly by TN cells when compared with other tg cells, and the influence of microenvironment generated during respective infection may all contribute to the observed differences. Varying affinity of peptide recognition by TCRs of TN and tg cells was shown by tetramer dissociation assays in an earlier work (Sehrawat et al., 2012). The possible contribution of differences in microenvironment and co-stimulatory molecules, as are expected to occur in a natural γ-HV infection, versus antigen delivery by other means, was not investigated further.

Multiple transcripts were clustered in different pathways such as cell cycle progression, apoptosis signaling, DNA replication, hypoxia response via hypoxia-inducing factors, oxidative stress response, T cell activation, the ubiquitin-proteasome pathway, and the p53 pathway (Figure 1E). A compilation of different biological processes represented by differentially expressed transcripts in ORF8 TN cells is shown in Tables S2 and S3. Genes encoding different classes of proteins that were differentially expressed by activated TN cells included nucleic acid-binding proteins, G-protein-coupled receptors, ribosomal proteins, transcription factors, ligand-gated ion channels, signaling molecules and adaptors, chaperones, growth factors, cell adhesion molecules, cytoskeletal proteins, splicing factors, extracellular matrix, as well as structural proteins (Figures S2A and S2B). Our RNA-seq data showed that transcripts for many genes were differentially expressed in activated TN cells associated with the effector functions of CD8^+^ T cells (Figures 1C and Table S1). We validated the altered expression of some of the genes differentially expressed in activated TN cells by cytofluorometry, and these include CD62L, TIM-3, PD1, CD44, CCR7, galectin-3, and IFN-γ at the protein product level (Figures 1B and S4A–S4D; Sehrawat et al., 2012).

#### Expression Profile of MHV68-Specific CD8^+^ T Cells upon Virus Infection

CD8^+^ T cells exponentially expand during the acute phase of infection and acquire a differentiation program that helps them migrate to the site(s) of infection to control infection (Kaech and Cui, 2012). Several genes differentially expressed by activated ORF8 TN cells represent such events (see Table S1 for a complete listing). In brief, transcripts for genes that encode for proinflammatory cytokines such as IFN-γ (up by 40-fold); granzymes B, A, and K (up by 300- to 900-fold); chemokine receptors such as CCR5 (up by 77 fold); and the chemokine RANTES (up by 300-fold) were highly upregulated in activated TN T cells when compared with their naive counterparts. Annexin II, a vesicular protein that facilitates extracellular transport of other proteins, was highly expressed (up by 250-fold) in activated TN cells, suggesting its critical role in effector functions of CD8^+^ T cells. Similarly, transcription factors that promote cellular proliferation and help produce several effector molecules were highly upregulated in expanded TN T cells. Some examples include Tbet (up by 93-fold), Zbtb32 (up by 67-fold), polycomb-group repressive complex 1 (up by 40-fold), and E2f2 (up by 35-fold). Other genes that are associated with activation and fate determination of CD8^+^ T cells such as *klrc1* (up by 54-fold), *lag3* (up by 48-fold), *klrg1* (up by 30-fold), *havrc2* (TIM-3: up by 30-fold), *pdcd1* (PD1: up by 26-fold), and *ctla4* (CTLA4: up by 17-fold) were also significantly upregulated in TN cells. Genes responsible for encoding Ca^++^-binding proteins such as those of the S100 family (*s100a6, s100a4, s100a9, s100a10, s100a8*) were upregulated up to 100-fold by activated TN T cells. Most of these proteins are involved in differentiation and cell cycle progression and are known to modulate tubulin polymerization (Donato et al., 2013). Genes encoding galectin-1 (*lgals1*: up by 87-fold) and galectin 3 (*lgals3*: up by 140-fold) were among the highly upregulated genes in activated TN T cells. In this study, we decided to investigate the role of galectin-3 in anti-γ-HV CD8^+^ T cell immunity, as will be described below.

Numerous genes were downregulated in virus-reactive activated ORF8 TN T cells compared with naive TN cells (Table S1). The extent of downregulation was more than 40-fold for five genes, between 20- and 40-fold for six genes, between 10- and 20-fold for approximately 40 genes, between 5- and 10-fold for more than 100 genes, and up to 2-fold for several thousand genes (Figures 1C and 1D and Table S1). One of the most downregulated genes in activated TN cells was *igfbp4* (down by 131-fold), which encodes insulin-like growth factor-binding protein 4. This molecule is involved in the differentiation of helper cells such as Th1, Th17, and regulatory T cells (Miyagawa et al., 2017). Whether or not this molecule plays a role in the differentiation of CD8^+^ T cells has not been investigated. Genes that encode for transporters of amino acids (*slc6a19:* down 42-fold, *slc6a5:* down 37-fold, *slc2a7*: down 11-fold) were highly downregulated in activated TN T cells compared with ion transporters (*slc4a1, slc7a1*), urea transporters (*slc14a1*), and carbohydrate transporters (*slc35b3*). This suggests that during the acute phase of a CD8^+^ T cell response, fate and function are greatly influenced by transport and metabolism of amino acids or by redox potential (Böhmer et al., 2005, Lin et al., 2015). The gene for the actin regulator FAM101B was also downregulated in activated TN cells. FAM101B localizes to the perinuclear space and helps stabilize perinuclear actin filament bundles (Gay et al., 2011). Its function in CD8^+^ T cell differentiation remains to be investigated. A gene *st6gal1,* which encodes ST6 β-galactoside α-2,6-sialyltransferase, was downregulated by 22-fold in activated TN cells, which might suggest a differential modification, particularly the capping of molecules such as CD45 with α-2,6-sialic acid during development of T cells in the thymus, when compared with their glycosylation profile during their HV-induced activation in the periphery (Elliott et al., 2018). Many such issues remain less well studied. The glycosylation status of different proteins in CD4^+^ T cells is known to control their differentiation program, but studies investigating its role in CD8^+^ T cell differentiation are limited (Toscano et al., 2007). Interleukin (IL)-7R (*il7r*: down by 25 fold) specifically marks precursors of memory CD8^+^ T cells during the acute phase of infection (Kaech et al., 2003). Similar to its expression pattern, genes for other molecules such as GPR114 (*gpr114*: down by 17-fold), IFN-γR2 (*ifngr2*: down by 16-fold), vasoactive intestinal peptide receptor 1 (*Vipr1*: down by 15-fold), interferon-inducible members of the *schlafen* family (*slfn5:* down by 15-fold), adhesion molecule with Ig-like domain 2 (*amigo2:* down by 14-fold), and TNF receptor superfamily member 25 (*tnfrsf25:* down by 14-fold) were among those downregulated in activated TN CD8^+^ T cells. Many of these molecules have been implicated in T cell differentiation, but the role of others remains to be explored (Geserick et al., 2004, Slebioda et al., 2011).

Apart from the genes described in this section, several thousand differentially expressed genes are listed in Tables S1 and S4.

#### Network Analysis for Significantly Differentially Expressed Genes during Activation of MHV68-Specific TCR-TN CD8^+^ T Cells

It is technically challenging to explore in depth all the genes whose expression changes significantly upon TN cell activation. Therefore, we performed a network analysis for those genes that were highly differentially expressed in naive and activated TN cells (Figure S3). In brief, the STRING network analysis revealed 229 nodes, which interacted with each other by 7,892 edges, and the average node degrees was 68.9. The average local clustering coefficient was found to be 0.721. A value of clustering coefficient nearing 0 suggests no clustering, whereas a value of 1 represents maximal clustering (Elliott et al., 2018). Many of the genes present in the network have been studied during differentiation of T cells expanded during infectious agents (Best et al., 2013, Wherry and Ahmed, 2004, Wherry et al., 2007). We focused our further analysis on the family of galectins that have critical role in immune responses during infection, autoimmunities, and cancers. We generated a STRING network for Lgals encoded by lgals genes (Figures S3B and S3D). Two such networks were obtained in which Lgals3 and Lgals1 served as hub genes. The network with Lgals3 revealed 10 interacting partners, whereas the one with Lgals1 revealed only six interacting partners each having a high protein protein interaction (PPI) enrichment score and p value less than 1.0 × 10^−16^ (Figures S3B and S3C). Lgals3 had more interacting partners and additionally included many partners of Lgals1. Given its critical role during activation of T cells, we chose galectin-3 for elucidating its role in CD8 T cell response induction during γ-HV infection (Figure S3D) (Hsu et al., 2009).

### Expression of Galectin-3 in Activated CD8^+^ T Cells

**Figure 2:**
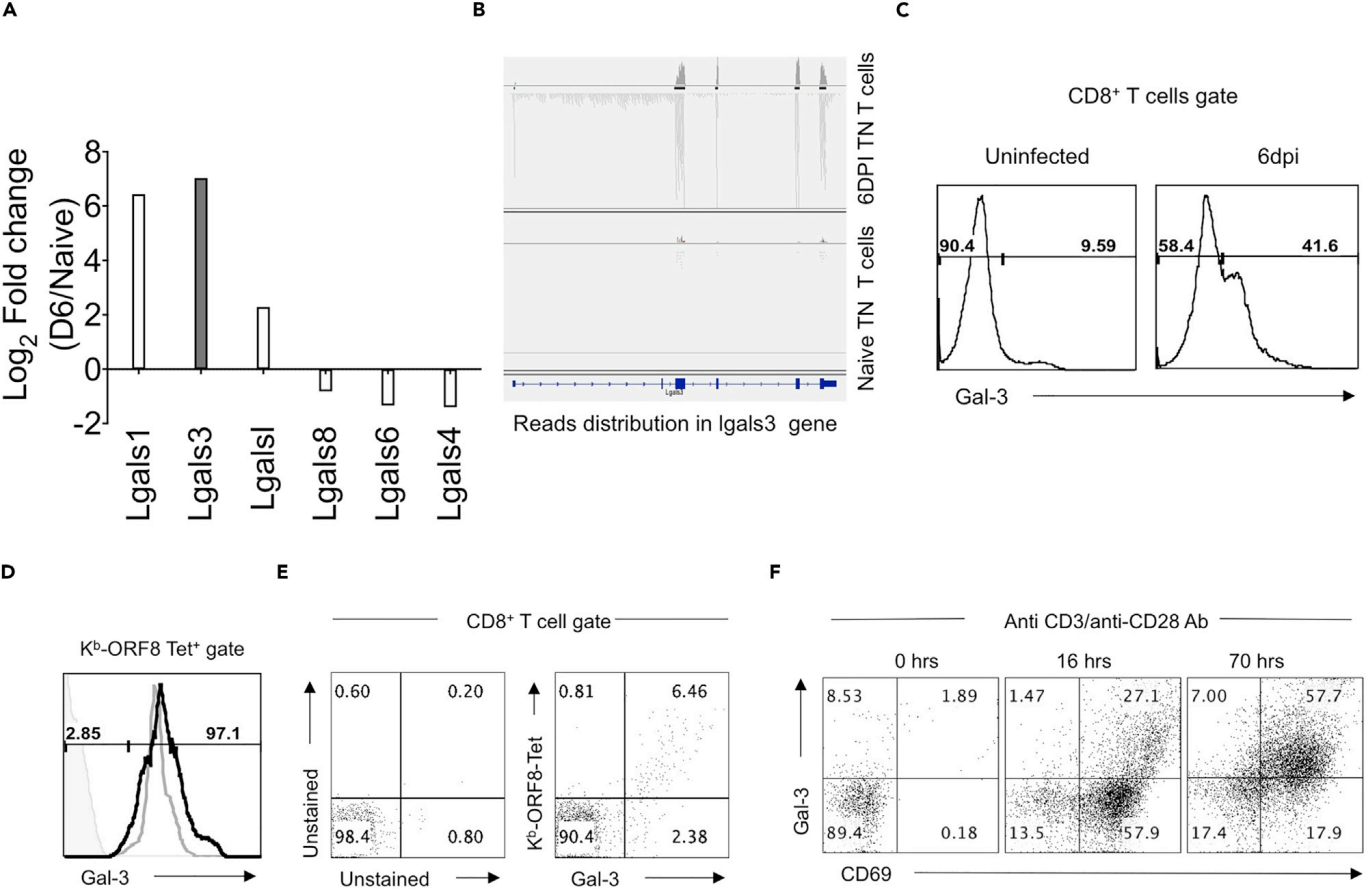
CD8^+^ T Cells Upregulate Galectin-3 Expression upon TCR and Co-receptor Ligation (A) Bar diagram shows a fold change in the expression of transcripts for different galectins in naive and activated TCR TN CD8^+^ T cells as a result of MHV68 infection as analyzed from RNA-seq data. (B) Integrative genomics viewers (IgV) snapshots to show the frequency of reads encompassing the exons (filled rectangles on the bottom line) versus intron (line without rectangles) of galectin-3 gene (Lgals3) in activated ORF8 TN CD8^+^ T cells (6 dpi) and their naive counterparts. (C) 50 × 10^3^ TN cells were transferred in congenic CD45.1 mice, and 6 days later the expression of galectin-3 was measured on total CD8^+^ T cells. (D) Galectin-3 expression was measured flow cytometrically in H-2K^b^-KNYIEFEKL-specific CD8^+^ T cells on 6 dpi to measure intracellular galectin-3 expression. The staining with specific antibodies followed Fc receptors blocking. Bold black line in overlaid histogram represents expression of galectin-3 in CD45.1^+^ cells (endogenous CD8^+^ T cells; ∼2% of total CD8^+^ T cells), and gray line represents galectin-3 expression in donor CD45.2^+^cells (TCR TN cells; 97.3% of total CD8^+^ T cells). (E) C57BL/6 mice were infected with MHV68, and CD8^+^ T cells from peripheral blood were analyzed for intracellular galectin-3 at 10 dpi. FACS plots show the expression of galectin-3 in K^b^-ORF8-tetramer-positive cells. The protocol used does detect cell surface and intracellular pool of galectin-3. (F) Purified CD8^+^ T cells were stimulated by soluble anti-CD3 (1 μg/mL) and anti-CD28 (1 μg/mL) in the presence of IL-2 in a round-bottomed plate for 16 and 70 hr. The expression of surface CD69 and intracellular galectin-3 was analyzed. Representative FACS plots are shown for all the experiments.

We analyzed and compared the transcriptional expression profile of all the members of galectin family in naive and the virus-activated ORF8 TCR TN CD8^+^ T cells (Figure 2A). Galectin-3 showed increased expression in activated TN T cells (up ∼140-fold) (Figures 2A and 2B). The expression of galectin-3 by polyclonal CD8^+^ T cells induced during γ-HV infection in the spleen is shown in Figure 2C. We also measured expression of galectin-3 protein in adoptively transferred TN cells and in endogenous CD8^+^ T cells that expanded in response to MHV68 infection in recipient congenic (CD45.1) mice (Figure 2D). A few naive CD8^+^ T cells (less than 10%) showed galectin-3 expression, whereas all virus-specific endogenous (∼2% of total CD8^+^ T cells) as well as donor TN CD8^+^ T cells (97.4% of total CD8^+^ T cells) scored positive for galectin-3 expression 6 dpi in spleen samples (Figure 2D). Endogenous MHV68-ORF8-specific CD8^+^ T cells were likewise analyzed for galectin-3 expression in the absence of an adoptive transfer. A large majority of H-2K^b^-ORF8-Tet^+^ CD8^+^ T cells in peripheral blood (Figure 2E) and spleen (data not shown) expressed galectin-3.

To investigate the kinetics of galectin-3 protein induction in CD8^+^ T cells, we stimulated sorted CD8^+^ T cells from WT mice with anti-CD3 and anti-CD28 antibodies. Intracellular and extracellular expression of galectin-3 was measured (Figures 2F and S4D). Upon activation, CD8^+^ T cells upregulated the intracellular pool of galectin-3 to a larger extent when compared with its surface expression (Figure S4D). More CD8^+^ T cells expressed intracellular galectin-3 (∼30% at 16 hr and more than 70% at 70 hr post stimulation) (Figure 2F). As expected, a large majority of CD8^+^ T cells were additionally upregulated CD69 upon stimulation (Figure 2F). The total galectin-3-expressing cells as detected in these assays are likely to include the cells expressing surface galectin-3 as well. Therefore, we measured its expression in both the permeabilized and intact cells (Figure S4D). These experiments revealed a predominant intracellular expression of galectin-3 in activated cells (Figure S4D).

### Galectin-3 Is Recruited at the Immunological Synapse during CD8^+^ T Cell Activation

**Figure 3:**
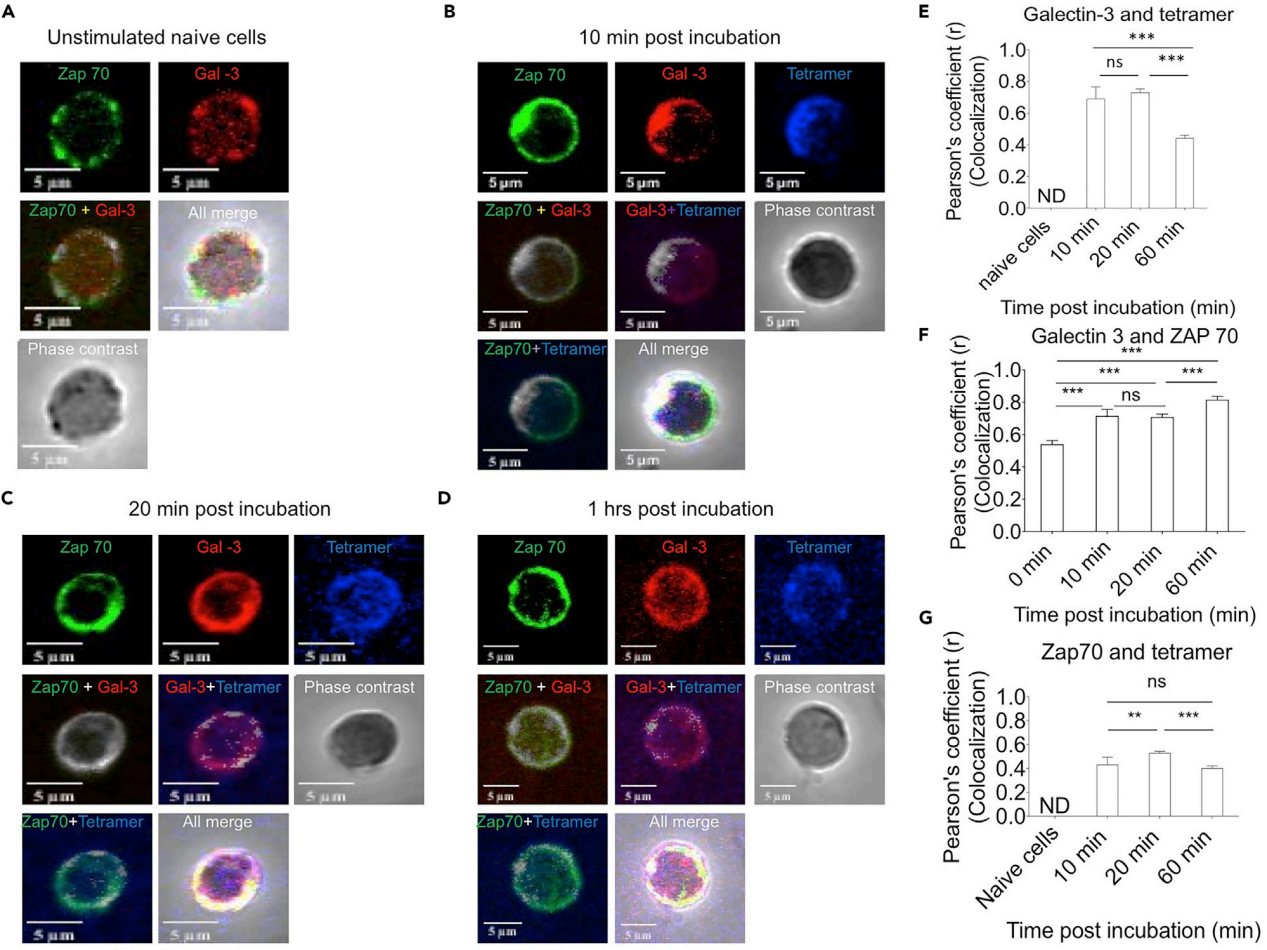
Galectin-3 Expressed by TCR-Stimulated CD8^+^ T Cells Is Recruited at Immunological Synapse Antigen-specific CD8^+^ T cells from OT1 mice were negatively selected by Magnetically activated cell sorting (MACS) purification and subsequently stimulated with specific peptide-loaded class I MHC tetramer (H-2K^b^-SIINFEKL-Tetramer-APC conjugated and immobilized on coverslips). Purified CD8^+^ T cells were added onto coverslips and incubated for varying times. At indicated time, cells were stained for different molecules, and confocal images were acquired as described in Transparent Methods. (A) OT1 cells incubated for 60 min on coverslips in the absence of H-2K^b^ tetramers were analyzed for different markers. Representative micrographs for individual staining and co-localization of Zap70, galectin-3, and tetramer are shown. (B–D) OT1 cells incubated for 10 min (B), 20 min (C), and 60 min (D) on coverslips in the presence of H-2K^b^ tetramers were similarly analyzed for different markers. Representative images for each group are shown. (E–G) Cumulative data for co-localization experiments obtained from more than 30 different cells is shown. Tukey multiple comparison test was used to determine the level of significance. Colocalization of galectin-3 and tetramer (E), galectin-3 and zap70 (F) and zap70 and tetramer (G) at different time points post incubation. ns, non-significant, ^∗∗^p <0.01 and ^∗∗∗^p<0.001.

During induction of CD8^+^ T cell responses, the TCR recognizes the p-MHC complex that is central to the immunological synapse. Other constituents are recruited subsequently. Most proteins that participate in the immune synapse are glycosylated. Galectin-3, through its CRD, could interact with them to influence T cell activation (Chen et al., 2009). We performed confocal microscopy to investigate whether galectin-3 expressed either at the basal level in CD8^+^ T cells or upregulated after stimulation of naive and memory CD8^+^ T cells is recruited to the immunological synapse. The extent of co-localization was measured and quantified as described (Dunn et al., 2011). After the TCR is ligated with cognate ligand, signaling events create docking sites for the cytosolic adapter molecule Zap70, which is recruited upon phosphorylation of ζ-chain of the co-receptor. We stimulated antigen-specific OT1 cells with immobilized H-2K^b^-SIINFEKL-tetramer-allophycocyanin and stained for galectin-3 and Zap70. Unstimulated cells served as controls. A diffuse and low level of expression for Zap70 was seen in naive cells (Figures 3A, 3E, and 3F). The extent of co-localization of galectin-3 with Zap70 was ∼50% in unstimulated cells. Within 10 min of incubation with MHC I tetramer, co-localization of galectin-3 with the MHC I tetramer increased (∼70 percent), as did galectin-3 with Zap70 (more than 70%) (Figure 3B). The extent of co-localization remained high at 20 min but began to diffuse at 60 min post stimulation (Figures 3C and 3D). Diffuse staining for Zap70 was apparent at 60 min (Figure 3D). Upon TCR stimulation, galectin-3 expressed at basal level in CD8^+^ T cells is co-localized with Zap70.

**Figure 4:**
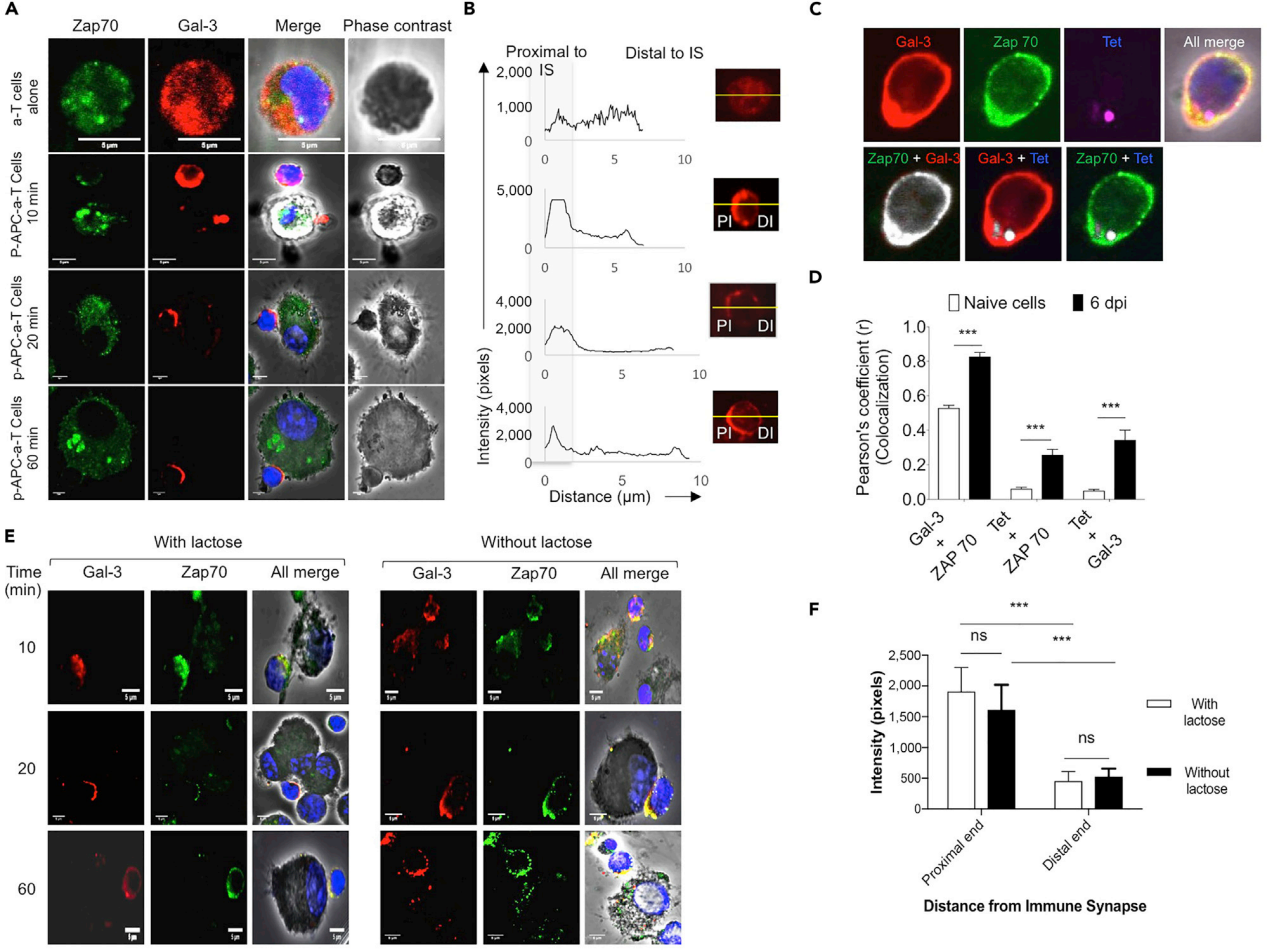
Galectin-3 Expressed by Re-stimulated CD8^+^ T Cells Is Recruited at Immunological Synapse Magnetically activated cell sorting (MACS)-purified OT1 cells were stimulated *in vitro* using anti-CD3 and anti-CD28 antibodies for 72 hr. Then cells were washed and co-cultured with SIINFEKL-peptide-pulsed BMDCs for indicated time. Thereafter cells were stained for different markers and analyzed by confocal microscopy for distribution of galectin-3. (A) Micrographs show the expression of different molecules and their migration toward immunological synapse involving activated OT1T cells and BMDCs at different time post stimulation. (B) The intensity of galectin-3 expressed in OT1 cells that migrated toward BMDCs is shown. Shaded area represents intensity of fluorescent probe proximal to immunological synapse (IS) (PIS) when compared with the intensity observed distal to immunological synapse (DIS). (C) OT1 cells were transferred in C57BL/6 mice 1 day before the infection with MHV68 M2-SIINFEKL. At 6 days post infection, H-2K^b^-SIINFEKL--tetramer-positive cells were FACS sorted and stained for galectin-3 and Zap70. Micrographs show staining of different molecules. (D) Bar graphs represent co-localization of different molecules in input control cells and OT 1 cells obtained from infected animals at 6 dpi. More than 35 cells were analyzed for each time point. ns, non significant, ^∗∗∗^ p<0.001. (E and F) The influence of α-lactose on the recruitment of intracellular versus extracellular galectin-3 at the immunological synapse was analyzed. SIINFEKL-peptide-pulsed BMDCs were co-cultured with OT1 cells FACS sorted from infected animals at 6 dpi in the presence or absence of 100 mM α-lactose solution, and the extent of localization of galectin-3 toward immune synapse was measured. A minimum of 35 cells was counted for each group for calculating co-localization percentages for different molecules. (E) Representative confocal micrographs for the co-localization of galectin-3 and Zap70 are shown. (F) Bar diagram depicting the intensity of galectin-3 at the proximal and distal ends of OT1 cells in contact with peptide-pulsed BMDCs for 35 cellular contacts for each sample is shown. ∗∗∗ p<0.001, ns, non significant.

Next, we investigated whether galectin-3 upregulated in previously stimulated CD8^+^ T cells is recruited to the immunological synapse upon their re-stimulation. To this end, OT1 cells were stimulated *in vitro* using anti-CD3 and anti-CD8^+^ antibodies for 72 hr. These cells were then co-cultured with SIINFEKL-pulsed Bone marrow derived dendritic cells (BMDCs) and stained for galectin-3 and Zap70. Cells not re-stimulated with peptide-pulsed BMDCs exhibited diffuse staining for galectin-3 (Figure 4A, upper panel). Within 10 min of co-culture with peptide-pulsed BMDCs, galectin-3 localized toward the immune synapse and remained so until 60 min; Figure 4A lower three panels and Figure 4B). In these experiments, we did not observe enhanced expression and recruitment of Zap70 to the synapse in re-stimulated cells. CD8^+^ T cells become temporarily refractory to re-stimulation as the TCR (Demotte et al., 2008). We also performed co-localization experiments using CD8^+^ T cells that were expanded as a result of viral infections and isolated in the acute phase of response at 6 dpi (Figures S5A–S5C). Galectin-3 was upregulated in antigen-specific CD8^+^ T cells responding to invading viral infections. We used two different viruses (a γ-HV [MHV68] and an influenza virus) to investigate galectin-3 expression and localization in reactive CD8^+^ T cells (Figures 4C, 4D, S5, and S6). Activated cells were then stained to assess co-localization of galectin-3 with class I MHC tetramers and Zap70. Staining of CD8^+^ T cells with class I MHC tetramers marked the expression of TCR. We observed significant co-localization of galectin-3 with MHC tetramers (∼40%) and Zap70 (more than 80%). In addition, Zap70 and class I MHC tetramers also exhibited a co-localization to the extent of 40% (Figures 4C and 4D). Therefore, our results show that galectin-3 is recruited at the immune synapse during activation of CD8^+^ T cells.

While analyzing co-localization of galectin-3 at immune synapse, we added α-lactose to the co-culture of activated OT1 cells and SIINFEKL-pulsed BMDCs to determine whether or not CD8^+^ T cells produced galectin-3 acts intracellularly or extracellularly. α-Lactose inhibits interaction between CRD of galectins and carbohydrate moieties present on other proteins (Demetriou et al., 2001). Addition of α-lactose, which is likely to act extracellularly by competing with galectins for binding to glycosylated proteins, did not alter the expression kinetics and recruitment of galectin-3 or Zap70 toward the proximal end of the immune synapse (Figures 4E and 4F). Whether or not α-lactose treatment of cells during their activation influences the activation of CD8^+^ T cells was also measured cytofluorometrically 12 hr post stimulation with plate-bound anti-CD3 and soluble CD28 antibodies (Figure S4E). CD8^+^ T cells stimulated in the presence of α-lactose displayed an impaired activation profile as measured by CD69 staining (Figure S4E). α-Lactose may act not only to compete with galectin-3 but also to other galectins. Therefore, we investigated the influence of extracellular galectin-3 neutralization by specific antibody clone (B2C10) that was previously shown to bind extracellular galectin-3 (Gordon-Alonso et al., 2017, Yip et al., 2017). We also performed experiments using this clone to validate its ability to bind cell surface as well as extracellular galectin-3 (Figure S4F). As naive and activated CD8^+^ T cells did not display significant amount of galectin-3 on their surface (Figure S4D), we reasoned that the primary immune cells other than those expressing CD8 molecule might be used to demonstrate its surface detection. Splenocytes as well as lymph node cells did not express galectin-3 on their surface, but about 25% of total splenocytes and 12% of lymph node cells that were CD8 negative show its surface expression (Figure S4F). In addition, we performed galectin-3 pull-down experiments using B2C10 clone of anti-galectin-3, which will demonstrate its ability to bind a soluble extracellular galectin-3. The results shown in Figure S4G demonstrate that a polypeptide of about 30 kDa, the molecular mass of galectin-3, was specifically bound by anti-galectin-3 antibody. The result of surface galectin-3 neutralization experiments using anti-galectin-3 antibodies are described in a later sections.

**Figure 5:**
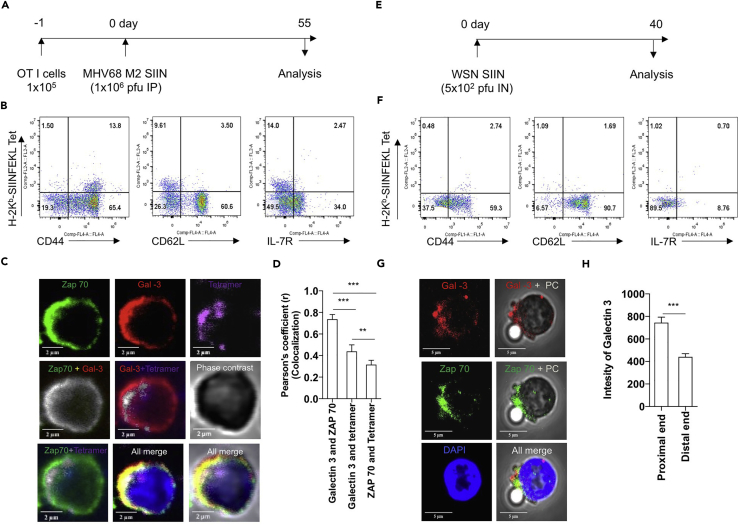
Galectin-3 Expressed by Memory Cells Generated during γ-HV (MHV-68 M2SIINFEKL) and Influenza Virus (WSN-SIINFEKL) during Their Recall Response Is Recruited at Immunological Synapse (A) 1 × 10^5^ OT1 cells were adoptively transferred in C57BL/6 mice 1 day before infection with MHV68-M2SIINFEKL virus. (B) Phenotypic characterization of H-2K^b^-SIINFEKL-specific CD8 T cells was performed flow cytometrically. Representative FACS plots are shown. (C and D) H-2K^b^-SIINFEKL-specific CD8 T cells were FCAS purified at 55 dpi and stimulated by H-2K^b^-SIINFEKL tetramer coated on coverslips for 1 hr. Thereafter cells were stained for different markers and analyzed by confocal microscopy for distribution of galectin-3. (C) Representative confocal micrographs show the expression of different molecules. (D) Bar graphs represent co-localization of different molecules in H-2K^b^-SIINFEKL cells. More than 30 cells were analyzed. ^∗∗^p<0.01 and ^∗∗∗^p <0.001. (E–H) (E) C57BL/6 mice were infected with WSN-SIINFEKL, and endogenous H-2K^b^-SIINFEKL-specific cells purified at 40 dpi by H-2K^b^-SIINFEKL-monomer-coated magnetic beads were analyzed by flow cytometry and confocal microscopy. (F) Phenotypic characterization of endogenous H-2K^b^-SIINFEKL-specific CD8^+^ T cells was performed flow cytometrically. Representative FACS plots are shown. (G) Representative confocal micrographs show the expression and co-localization of different molecules. (H) Bar diagram shows the intensity (pixels) of galectin-3 at the proximal and distal ends of specific cells and H-2K^b^-monomer-coated bead contacts. At least 30 such contacts were counted. ^∗∗^ p value < 0.01 and ^∗∗∗^ p value < 0.001.

Long-term protective immunity to intracellular infections or vaccines is critically dependent on memory response. Therefore, we investigated whether or not galectin-3 is also involved in regulating the immune synapse formation by virtue of its localization at proximal end during the activation of memory CD8^+^ T cells. We infected OT1-cell-recipient mice with MHV68-SIINFEKL and analyzed CD8^+^ T cells in the memory stage (55 dpi) by flow cytometry and confocal microscopy (Figure 5A). All H-2K^b^-SIINFEKL-specific CD8^+^ T cells expressed high level of CD44 (CD44^hi^) but low level of CD62L (CD62L^lo^) and IL-7R (IL-7R^lo^) (Figure 5B). This phenotype was previously reported during a persistent γ-HV (MHV68) infection (Jennings et al., 2014). Galectin-3 co-localized with Zap70 and tetramers to the extent of ∼80% and ∼40%, respectively (Figures 5C and 5D), whereas co-localization of Zap70 with tetramers was ∼30% in stimulated H-2K^b^ SIINFEKL-specific cells with immobilized tetramers. Therefore, similar to what was observed during the initial activation of naive CD8^+^ T cells after MHV68 infection, the stimulation of specific memory CD8^+^ T cells exhibited galectin-3 recruitment at the immune synapse.

The phenotype of memory CD8^+^ T cells generated during a persistent infection is distinct from that generated during an acute viral infection. We therefore analyzed memory cells generated during an acute infection with influenza virus. We infected mice with an influenza virus encoding a SIINFEKL epitope (WSN-SIINFEKL) and analyzed endogenous antigen-specific memory CD8^+^ T cells at 40 dpi (Figure 5E). Infected mice demonstrated a transient drop in body weight (Figure S5B). H-2K^b^-SIINFEKL-specific CD8^+^ T cells isolated in the acute phase of response demonstrated a high expression of activation marker CD44 and KLRG1 (Figure S5C). When analyzed during memory stage after 40 dpi, H-2K^b^-SIINFEKL-specific CD8^+^ T cells were heterogeneously stained for CD44, CD62L, and IL-7R, a phenotype distinct from those of memory cells generated during persistent MHV68 infection (Figure 5F). Sorted H-2K^b^-SIINFEKL-specific CD8^+^ T cells during the acute (Figure S5D) as well as memory stage (Figures 5G and 5H) of infection were analyzed for recruitment of galectin-3 at the immune synapse. As shown in Figures S5D, 5G, and 5H, galectin-3 was recruited alongside Zap70 at the synapse formed between SIINFEKL-specific CD8^+^ T cells and H-2K^b^-SIINFEKL-coated beads.

Taken together, our results demonstrate that galectin-3 is upregulated in antigen-specific CD8^+^ T cells upon MHV68 as well as influenza virus infection and is recruited to the immunological synapse during their primary as well as memory stimulation.

### Galectin-3 Regulates Proliferation and Cytokine Production by CD8^+^ T Cells

**Figure 6:**
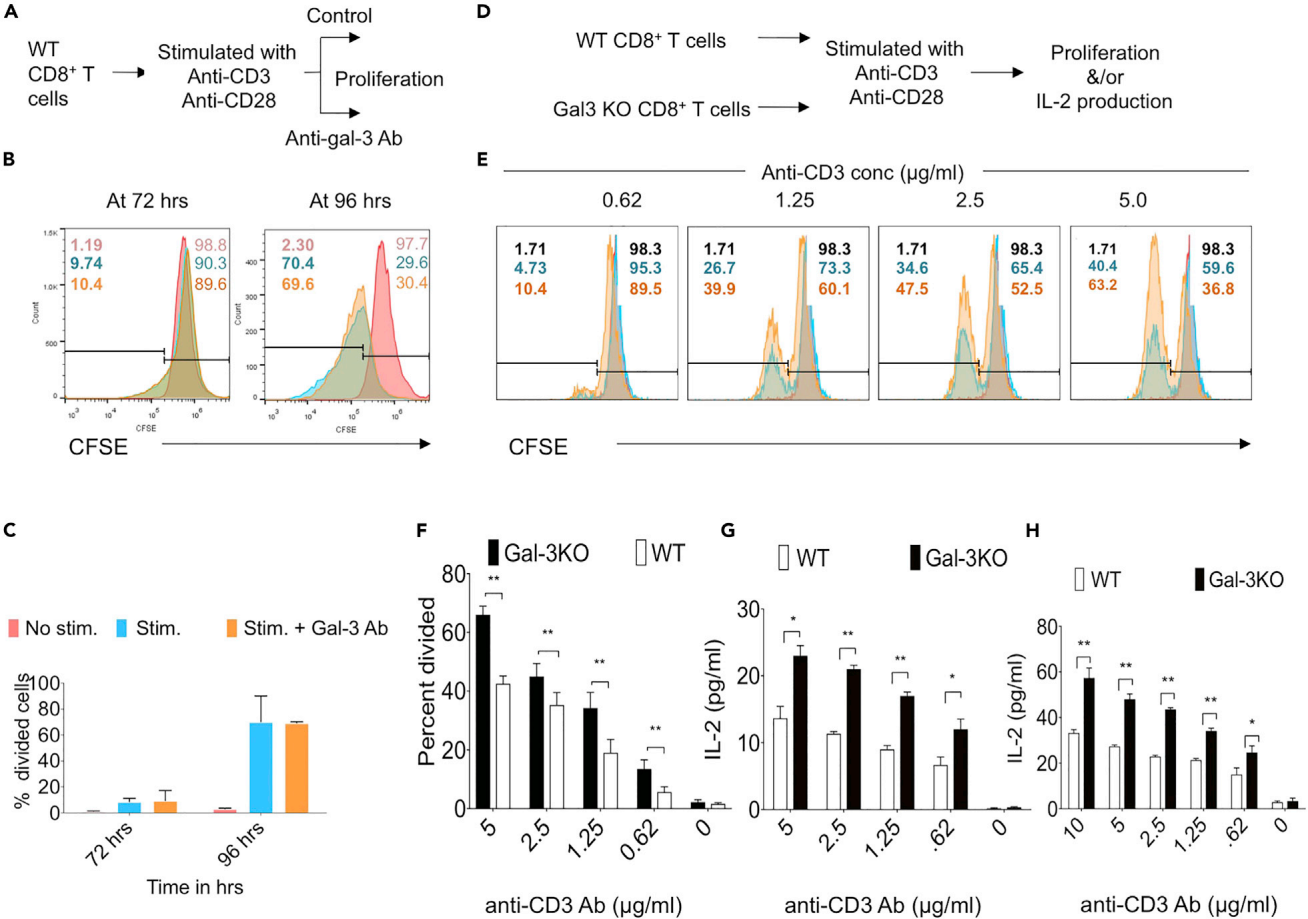
Cell-Autonomous Intracellular Expression of Galectin-3 in CD8^+^ T Cells Is Responsible for Regulating Their Proliferation and Cytokine Production (A) A schematic of the experiment to investigate influence of galectin-3 in anti-MHV68 specific CD8^+^ T cell responses. Purified CD8^+^ T cells labeled with CFSE were stimulated with plate bound anti-CD3 (1μg/ml) and soluble anti-CD28 (1μg/ml) antibodies in the presence or absence of neutralizing anti-galectin-3 (10μg/ml) antibody. The proliferation was measured by CFSE dilution. (B) Representative overlaid histograms show the proliferation of CD8^+^ T cells in the presence or absence of anti-galectin-3 antibody. The numbers written in bold fonts represent the numbers of divided cells in unstimulated cells (reddish), stimulated cells (blue) and stimulated cells added with anti-galectin-3 antibody (orange). (C) Bar diagram show the percentage of divided cells at 72 hrs and 96 hours in CD8^+^ T cells stimulated in the presence or absence of anti-galectin-3 antibody. Addition of neutralizing gal-3 antibody did not alter the proportion of divided cells. D-H. An influence of galectin-3 in causing CD8^+^ T cell activation was measured using gal-3 KO T cells. (D) A schematic of the experiment is shown. CD8^+^ T cells were MACS purified from WT and galectin-3 KO mice and labeled with CFSE. Labeled cells were then stimulated in vitro with indicated concentration of soluble anti-CD3 and 1μg/ml of anti-CD28. Dilution of CFSE in CD8^+^ T cells was measured cytofluorimetrically and IL-2 levels were measured in the culture supernatants by sandwich ELISA. (E) Histograms show the proliferation of WT and galectin-3 deficient CD8^+^ T cells at indicated concentrations of anti-CD3. The numbers written represent the proportion of divided cells (on left side) and undivided cells (right side of histograms) in un-stimulated (black), WT (blue) and galectin-3 deficient CD8^+^ T cells (orange). (F) Bar diagram show the percentage of divided cells from galectin-3 KO and WT animals when stimulated with indicated concentrations of anti-CD3. ^∗∗^p < 0.01. (G) Bar diagrams show IL-2 levels in the culture supernatants of WT and galectin-3 deficient CD8 T cells. ^∗^ p < 0.05, ^∗∗^p < 0.01. (H) Total splenocytes isolated from WT and galectin-3 KO animals were stimulated with indicated concentrations of anti-CD3 and 1μg/ml of anti-CD28 for 60 hours and IL-2 levels were measured by ELISA. Bar diagrams show the levels (mean±SD) of IL-2 for three separate wells in WT and galectin-3 KO animals at indicated concentration of anti-CD3. ^∗^p < 0.05, ^∗∗^p < 0.01. The experiments were repeated three times with similar results.

Having established that galectin-3 is recruited to the immunological synapse in CD8^+^ T cells after stimulation, we investigated whether extracellular galectin-3 neutralization improves activation and cytokine production by CD8^+^ T cells *in vitro*. We stimulated purified Carboxyfluorescein succinimidyl ester (CFSE)-labeled CD8^+^ T cells with anti-CD3 and soluble anti-CD28 antibodies in the presence or absence of a neutralizing anti-galectin-3 antibody (Figure 6A). In the absence of antibody treatment, approximately 10% and 70% of CD8^+^ T cells divided at 72 and 96 hr post stimulation, respectively, upon incubation with anti-CD3 (1 μg/mL of plate-bound) and soluble anti-CD28 antibodies (Figures 6B and 6C). Extracellular galectin-3 neutralization with neutralizing antibody did not affect the proportion of divided cells as measured by CFSE dilution assays at 72 and 96 hr post stimulation (Figures 6B and 6C).

To further investigate a cell-autonomous contribution of galectin-3 in activating CD8^+^ T cells, we magnetically isolated CD8^+^ T cells from WT and galectin-3 KO mice and stimulated equal numbers of CFSE-labeled CD8^+^ T cells, by application of different concentrations of anti-CD3 and soluble anti-CD28 (1 μg/mL) antibodies. We measured CFSE dilution as an indicator of proliferation and IL-2 production in culture supernatants as an indicator of their functionality (Figure 6D). More CD8^+^ T cells from galectin-3 KO mice proliferated than those from WT mice at all concentrations of anti-CD3 antibody tested (Figures 6E and 6F). Galectin-3-deficient CD8^+^ T cells produced more IL-2 than WT CD8^+^ T cells in response to anti-CD3/anti-CD28 antibody treatment (Figure 6G). We also stimulated splenocytes from galectin-3 KO and WT mice with anti-CD3/anti-CD28 antibodies and measured their proliferation and cytokine production. Similar to the response of CD8^+^ T cell, total T cells, which also included responding CD4^+^ T cells from galectin-3 KO mice, produced more IL-2 than their WT counterparts (Figure 6H).

A deficiency of galectin-3 in T cells is therefore responsible for their enhanced proliferation and cytokine production, and galectin-3 could predominantly act in a cell-autonomous manner and intracellularly to inhibit T cell functions.

### Galectin-3 Deficiency Enhances MHV68-Specific CD8^+^ T Cell Response

**Figure 7:**
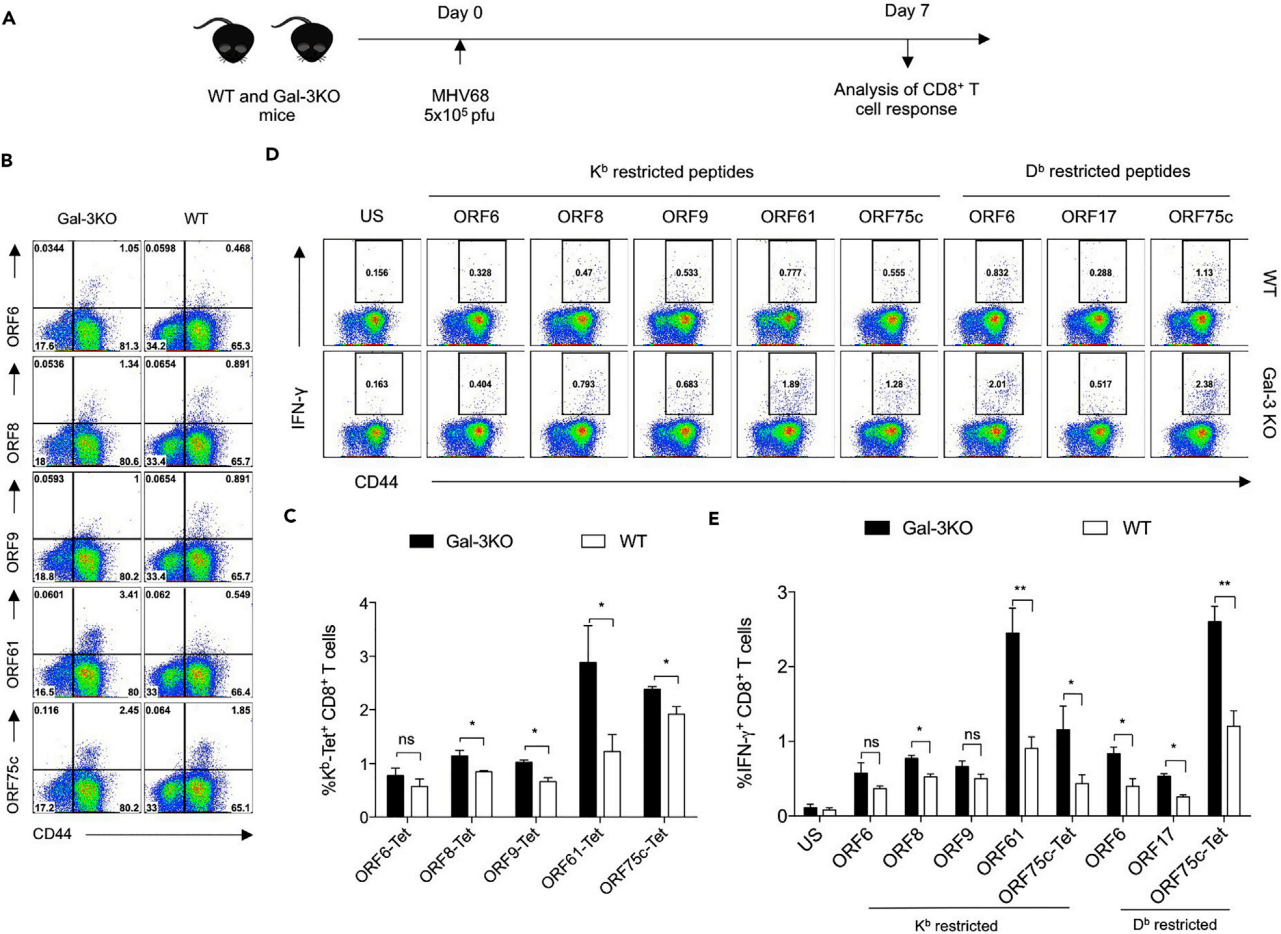
Galectin-3 Deficiency Enhances Magnitudes of γ-HV-specific CD8^+^ T Cells (A) A schematic of the experiment to investigate influence of galectin-3 in anti-MHV68-specific CD8^+^ T cell responses. Galectin-3 KO and C57BL/6 WT mice were i.p. infected with MHV68, and splenocytes were analyzed for surface TCR expression and intracellular cytokine staining. (B) Representative FACS plots show the frequencies of respective tetramer-specific cells in spleen samples of infected WT and galectin-3 KO mice. Tetramers for respective ORFs were generated using a UV-mediated photocleavage reaction, and the exchange was performed to displace conditional ligand with peptide sequences derived from the indicated ORFs of MHV68. (C) Bar diagrams show the frequencies of respective peptide-specific CD8^+^ T cells measured from at least four different animals per group. The experiments were repeated at least two times with similar results. (D) Representative FACS plots show IFN-γ-producing CD8^+^ T cells measured using ICCS assays. Splenocytes from WT and galectin-3 KO animals were stimulated with peptides derived from indicated ORFs of MHV68, and the cells were analyzed using flow cytometry. (E) Bar diagrams show the frequencies (mean ± SD) of IFN-γ-producing cells reactive to class I MHC epitopes of indicated ORF-derived peptides measured for four WT and galectin-3 KO animals per group.

After demonstrating a regulatory intracellular function of galectin-3 during the immunological synapse formation, we explored whether a loss of galectin-3 expression has functional consequences in modulating a virus-specific CD8^+^ T cell response. We infected WT and galectin-3 KO mice with MHV68 (Figure 7A) and measured the magnitude of virus-specific CD8^+^ T cells isolated from spleens by MHC tetramer staining and ICCS assays. Galectin-3 KO animals mounted a stronger virus-specific CD8^+^ T cell response than WT animals (Figures 7B–7E). Using MHC tetramers, we investigated recognition of CD8^+^ T cell epitopes derived from five different ORFs of MHV68. Both early (ORF9 and ORF75c) and late (ORF6, ORF8, ORF17, and ORF61) MHV68 antigens were examined (Freeman et al., 2010, Gredmark-Russ et al., 2008). Galectin-3 KO mice showed greater expansion of their virus-specific CD8^+^ T cells than WT mice (Figures 7B and 7C). Intracellular cytokine assays were performed to measure IFN-γ-producing cells in response to H-2K^b^- and H-2D^b^-restricted peptides. The frequencies of cytokine-producing splenic CD8^+^ T cells were significantly higher for most of the epitopes for galectin-3 KO animals than those for WT animals (Figures 7D and 7E). ORF61- and ORF75c-reactive CD8^+^ T cells expanded to a greater extent than those recognizing other ORFs (Figure 7). CD8^+^ T cells that recognize ORF61, a ribonucleotide reductase, may be critically involved in the maintenance of viral latency (Freeman et al., 2010, Gredmark-Russ et al., 2008). ORF75c, a tegument protein of MHV68, exhibits ubiquitin E3 ligase activity and can target degradation of promyelocytic leukemia, essential for the pathogenesis of γ-HV-induced transformation and in modulating apoptosis (Ling et al., 2008). An ORF75c-null virus is compromised in its ability to establish latency as well (Gaspar et al., 2008). Thus, enhancing the frequency of CD8^+^ T cells specific for ORF75c and ORF61 may be a means of interfering with γ-HV-mediated pathogenesis. Lack of galectin-3 signaling in virus-specific CD8^+^ T cells by enhancing their responsiveness could perhaps improve protection against this γ-HV.

### Enhanced MHV68-Specific CD8^+^ T Cell Response Leads to More Efficient Viral Control in Galectin-3 KO Mice

**Figure 8:**
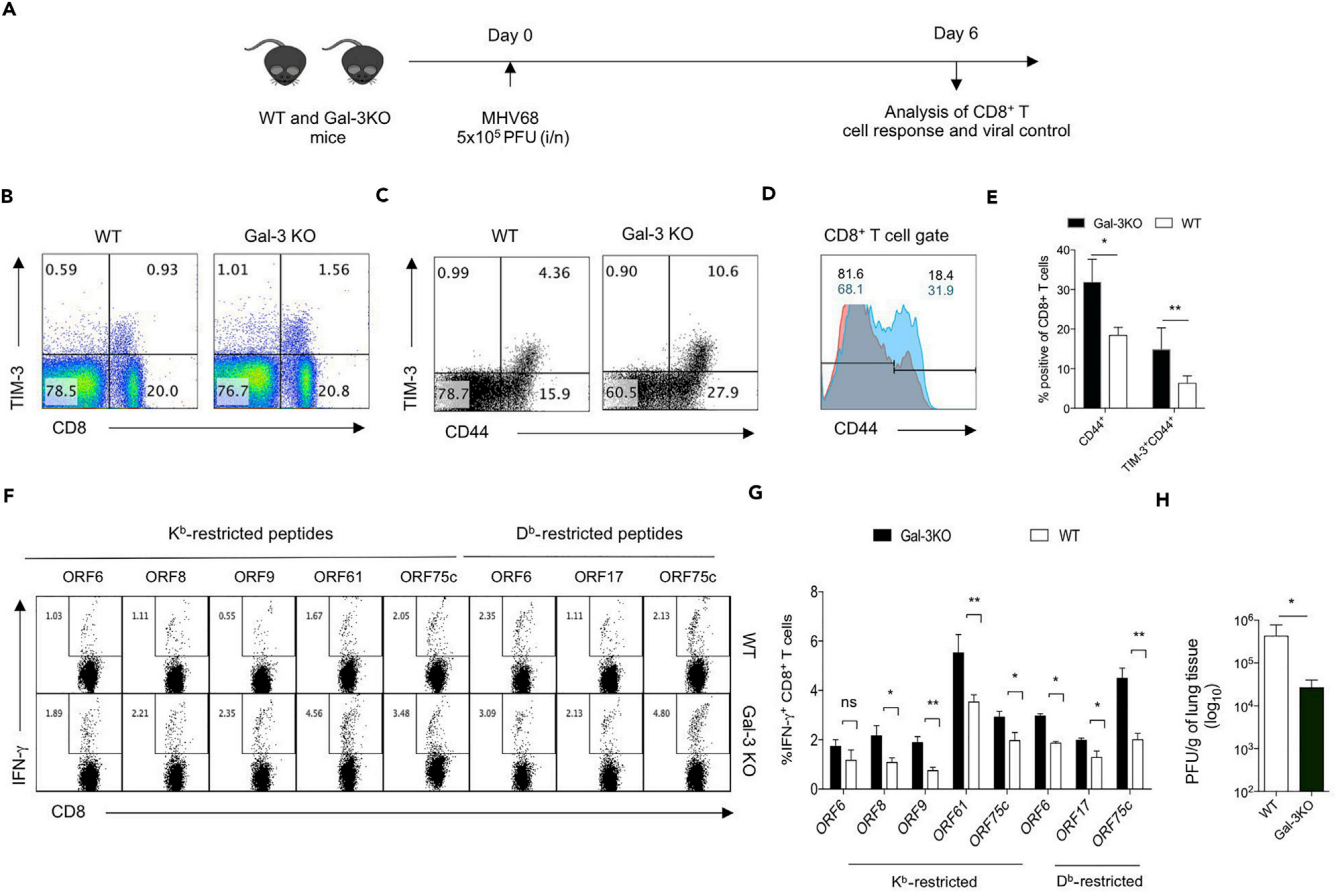
Galectin-3-Deficient Animals Efficiently Control γ-HV Infection (A) A schematic of the experiments is shown. Galectin-3 KO and C57BL/6 WT mice were intranasally infected with 5 × 10^5^ plaque-forming unit (PFU) of MHV68. Mediastinal lymph nodes were analyzed at 6 dpi by surface staining and intracellular cytokine staining for measuring the phenotype and functions of CD8^+^ T cells. Lungs were collected from the infected animals, and the viral titers were measured. (B) Representative FACS plots show the frequencies of TIM-3^+^-activated CD8^+^ T cells in live cell gate isolated from the lymph nodes of WT and galectin-3 KO mice. (C) FACS plots of CD8^+^ T cell-gated population show the expression of CD44 and TIM-3 in CD8^+^ T cells from WT and galectin-3 KO mice. (D) Histograms show the expression of CD44 in gated CD8^+^ T cells isolated from lymph nodes of WT and galectin-3 knockout mice. Numbers in overlaid histograms represent proportion of CD44^lo^ and CD44^hi^ populations of WT (regular font) and galectin-3 knockout (bold font). (E) Bar diagrams show the percentage of CD44^+^ and CD44^+^TIM-3^+^ CD8^+^ T cells in WT and galectin-3 KO animals. ^∗^p<0.05, ^∗∗^p <0.01. (F) FACS plots show the frequencies of IFN-γ-positive cells in response to different MHC class I (H-2K^b^ and H-2D^b^-restricted) epitopes derived from ORFs of MHV68. (G) Bar diagrams showing the frequencies (mean ± SD) of IFN-γ-producing CD8^+^ T cells from a representative experiment in which four WT and four galectin-3 KO animals were used. ns, non significant, ^∗^ p< 0.05, ^∗∗^ p< 0.01. (H) Bar diagram shows virus titers in the lungs of the four animals each from WT and galectin-3 KO group at 6 dpi. The experiments were repeated two times with similar results. ^∗^p <0.05.

We asked whether or not a stronger anti-viral CD8^+^ T cell response in galectin-3 KO mice would help control the virus more efficiently than in WT mice. Galectin-3 KO and WT animals were infected intranasally with MHV68 so that we could measure replicating virus in lung tissues. The functionality of anti-viral CD8^+^ T cells and viral titers in lung tissues of infected animals were then measured (Figure 8A). We measured the frequencies of activated CD8^+^ T cells, i.e., TIM-3^+^, CD44^+^, and TIM-3^+^CD44^+^ CD8^+^ T cells, in the mediastinal lymph nodes of MHV68-infected galectin-3 KO and WT mice (Figures 8B–8E). TIM-3 expression on CD8^+^ T cells marks antigen-experienced cells (Sehrawat et al., 2010). More TIM-3^+^CD8^+^ T cells were present in the lymph node (LN) of galectin-3 KO animals (10.6%) than in WT animals (4.6%) (Figures 8B–8E). Only a subset CD44^hi^CD8^+^ T cells expressed TIM-3 (Figure 8C). Galectin-3 KO animals also had more activated CD8^+^ T cells (CD44^hi^ CD8^+^: 18% in WT and 31% in galectin-3 KO mice) (Figures 8D and 8E). The frequencies of IFN-γ-positive CD8^+^ T cells isolated from MLN were measured by ICCS assays in response to known MHV68-specific CD8^+^ T cell targets. Seven of these eight peptides evoked significantly more IFN-γ-positive CD8^+^ T cells in galectin-3 KO animals (Figures 8F and 8G). This was true for both H-2K^b^- and H-2D^b^-restricted epitopes. Viral titers were measured in lung tissues of galectin-3 KO and WT mice and showed up to a 100-fold reduction in galectin-3 KO mice on 6 dpi (Figure 8H). Similar results were obtained when viral titers were measured at different time points (data not shown).

### Galectin-3 Contributed by Cells Other Than CD8^+^ T Cells Does Not Affect Virus-Induced Expansion of Specific CD8^+^ T Cells

**Figure 9:**
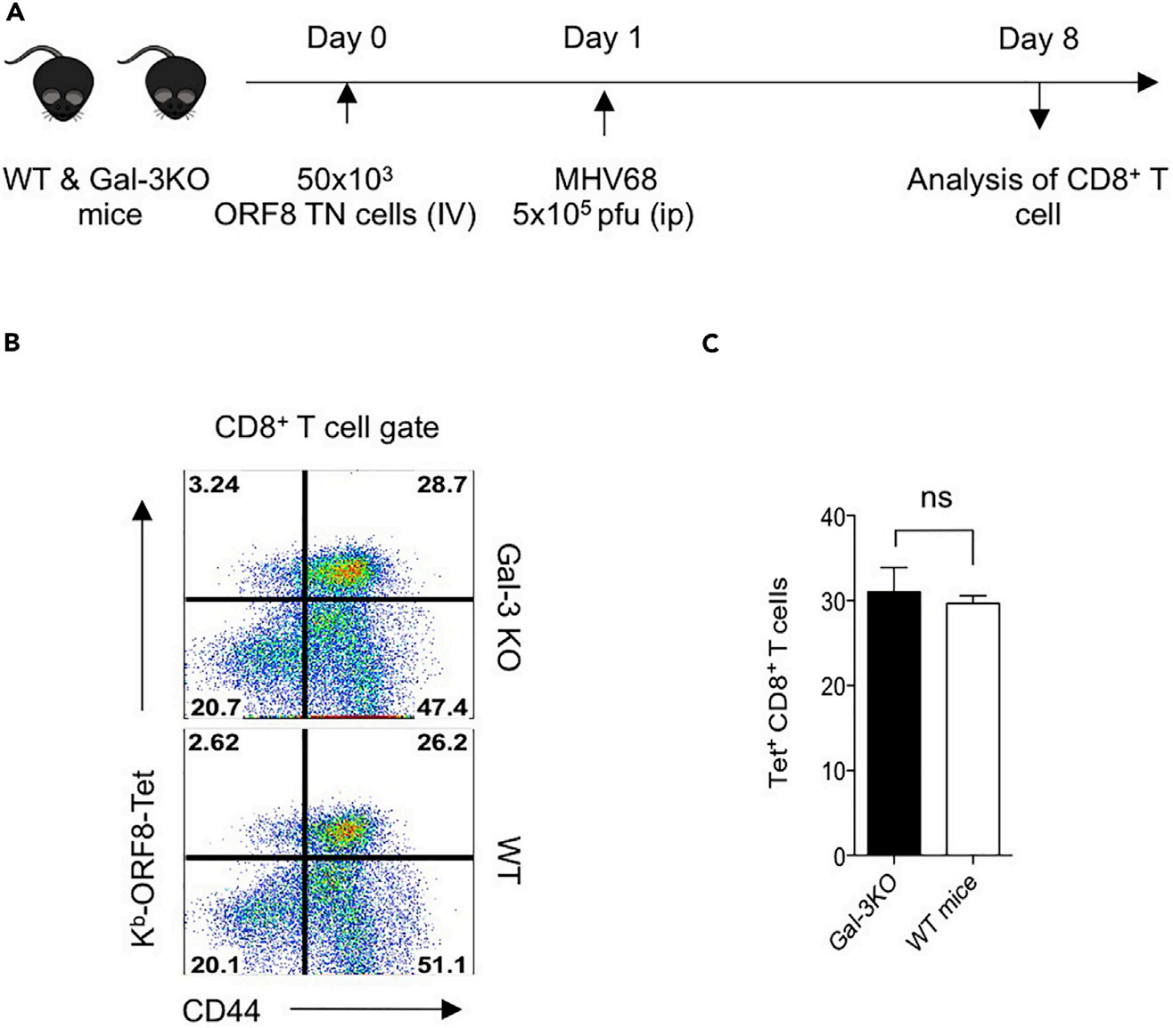
Expansion of Virus-Specific CD8^+^ T Cells during Acute Phase of Infection Is Not Influenced by Exogenous Galectin-3 (A) A schematic of the experiments is shown. 50 × 10^3^ ORF8 TN cells were adoptively transferred into WT and galectin-3 KO animals, which were then i.p. infected with MHV68. At 6 dpi, the frequencies of ORF8-tetramer-positive cells were analyzed. (B) Representative FACS plots show the frequencies of K^b^-ORF8-tetramer-positive cells in the spleen samples of animals. (C) Bar diagrams show the frequencies (mean ± SD) of H-2K^b^-ORF8 CD8^+^ T cells obtained from three WT and galectin-3 KO animals. The experiments were repeated two times with similar results. ns, non significant.

Many cell types, including T cell, B cells, myeloid cells, and stromal cells may produce galectin-3 (Hsu et al., 2009). We investigated whether or not galectin-3 contributed by cells other than CD8^+^ T cells affects the expansion of virus-specific CD8^+^ T cells. We transferred 50 × 10^3^ ORF8 TCR TN CD8^+^ T cells into WT and galectin-3 KO mice 1 day before MHV68 infection and measured the frequencies of expanded cells 7 days later (Figure 9A). Both endogenous and transferred ORF8-specific TN cells were analyzed. The proportion of activated and expanded ORF8 TCR TN CD8^+^ T cells was similar in both the galectin-3 KO and WT animals (Figures 9B and 9C), suggesting a negligible effect of exogenous galectin-3 in the magnitude of γ-HV-specific CD8^+^ T cells. Galectin-3 deficiency therefore may exert a cell-autonomous phenotype in CD8^+^ T cells expanded during MHV68 infection.

## Discussion

γ-HVs are species-specific pathogens. An investigation of their pathogenesis thus requires a natural host as the relevant animal model. An understanding of host correlates of protection to γ-HVs is confounded by the likelihood that these may show host-specific elements as well. Consequently, strategies for vaccine development have suffered due to the absence of reliable animal models. MHV68 infection of mice offers one such model system to study the immunity and immunopathology to γ-HVs (Barton et al., 2011). We generated a CD8^+^ TCR TN mouse model that serves as a source of homogeneous virus-specific CD8^+^ T cells, which recognize an epitope derived from membrane glycoprotein B of MHV68 (Sehrawat et al., 2012). We performed RNA-seq on naive and activated MHV68-specific CD8^+^ TCR TN T cells, the latter isolated in the acute phase of infection, to obtain insights into the molecules and pathways that may be critical to their function. Genes for many transcripts encompassing different biological processes, cellular components, and classes of proteins and of diverse molecular function were differentially expressed. Many of these transcripts have not been reported previously to exhibit a similar expression pattern in the acute phase of other viral infections (Best et al., 2013, Wherry et al., 2007). Further analysis showed that more than 300 transcripts in our dataset were not mapped to the existing Gene Ontology database (Table S5). The transcriptional analysis of endogenous CD8^+^ T cells is commonly confounded by representation of innumerable TCR affinities, as the analyzed cell population is heterogeneous. The transcriptomic data on differentiating single cell is limited but could provide valuable information. RNA-seq data of differentiating TN CD8^+^ T cells could provide further insight into their phenotypic and functional attributes. These RNA-seq data could uncover new angles of attack to investigate CD8^+^ T cell biology. Of course, not only transcription but also the translational status determines the fate of differentiating CD8^+^ T cells (Araki et al., 2017). Numerous genes differentially expressed in activated TN CD8^+^ T cells suggest that during differentiation of T cells the translational machinery may be impaired, which could contribute to the contraction of almost 95% of antigen-reactive CD8^+^ T cells. Manipulation of the translational profile of antigen-stimulated cells could modulate immunological memory and control of infection (Sehrawat et al., unpublished data).

In response to an invading intracellular pathogen, CD8^+^ T cells expand massively to resolve the infection. Most of the expanded pathogen-specific effector cells are then eliminated during the contraction phase (Ahmed and Gray, 1996). However, mechanisms responsible for their induction, expansion, and elimination are still not entirely understood and may show considerable heterogeneity, depending on the biology of the pathogen recognized. The role of galectins in the pathogenesis of infection is beginning to be understood (Vasta, 2009). Galectins play a diverse role in immunity upon infection, in autoimmune diseases and cancer (Rabinovich and Toscano, 2009). Through direct interactions with the pathogen or by modulating the microenvironment in which pathogens reside, galectins can either promote or prevent viral infections (Vasta, 2009). For example, galectin-1 could promote HIV infection of macrophages by directly binding to its surface glycoproteins and serving as a bridge between the virus and immune cells (Mercier et al., 2008, Ouellet et al., 2005). Galectins can also help attract susceptible immune cells to sites of infection (Sano et al., 2000). Viruses such as HIV, EBV, Nipah virus, and HV 1 modulate the expression of various galectins in host cells (Vasta, 2009). One could therefore argue that galectin-3 deficiency might interfere with a productive replication of γ-HVs such that the overall antigen levels are reduced. We observed enhanced anti-viral CD8^+^ T cell immunity in galectin-3 KO mice, which could represent an underestimation rather than an overestimation of antigen-specific CD8^+^ T cells. Galectin 3-deficient animals mounted a stronger anti-MHV68 CD8^+^ T cell response to seven of eight epitopes, regardless of whether restricted by H-2K^b^ or H-2D^b^, thus improving virus control. Similarly an enhanced expansion and cytokine production by co-receptor-stimulated CD8^+^ T cells was observed.

During activation of CD8^+^ T cells, actin microfilaments help coalesce the glycoproteins involved in the formation of immunological synapse (Dustin and Cooper, 2000). Our RNA-seq data showed upregulation of two members of the galectin family, i.e., galectin-3 (up ∼140-fold) and galectin-1 (up ∼87-fold), in activated TN CD8^+^ T cells during the acute phase of infection with MHV68. Galectin-3 has one CRD but exhibits a greater tendency to multimerize upon binding to glycoconjugates than other galectins (Lepur et al., 2012). This effect is attributed to its non-lectin N-terminal domain, which is rich in proline and glycine residues (Menon and Hughes, 1999). Exposure of T cells to exogenous galectin-3 might cause their apoptosis, whereas endogenous expression of galectin-3 was found to exert an anti-apoptotic function (Fukumori et al., 2004). However, another study showed that activated CD8^+^ T cells as a result of herpes simplex virus 1 did not undergo apoptosis upon treatment with exogenous galectin-3 (Sehrawat et al., 2010). Therefore, the influence of galectin-3 on immune cells could be context dependent.

Galectin-3 is recruited to the immunological synapse during activation of CD4^+^ T cell response (Chen et al., 2009). In view of its upregulation in MHV68-specific TCR TN CD8^+^ T cells, we investigated whether galectin-3 expression has a functional consequences in resolving the infection. We find that galectin-3 expression was upregulated at the transcriptional and at a protein product level upon viral infections as well in co-receptor-stimulated CD8^+^ T cells within 16 hr, and the expression further increased on 3 days post stimulation (Figure 2). This was true for MHV68- as well as influenza-virus-expanded antigen-specific CD8^+^ T cells, thereby attesting to the fact that pathogen-specific elements and their interaction by host could play a role in fine-tuning T cell responses (Figures 5 and S5). The milieu generated during some chronic infection is enriched in inhibitory cytokines such as IL-10, which could promote the activity of glycosyltransferases such as Mgat5, which in turn promoted the modification of N-linked glycans for various surface proteins (Smith et al., 2018). Galectin-3 might interfere with the recruitment of such proteins during activation of T cells, thereby increasing the threshold of TCR signaling. This effect may be predominantly extracellular in nature. Our investigation focusing on intracellular versus extracellular involvement of galectin-3 in shaping T cell responses against γ-HVs revealed predominantly an intracellular role of this molecule as the neutralization of extracellular galectin-3 either by α-lactose or by anti-galectin-3 antibody did not seem to influence the recruitment of galectin-3 to immune synapse or the proliferation of stimulated cells (Figures 4 and 6). The α-lactose treatment could also interfere with the activity of other galectins, which was also reflected in experiments investigating its role in the activation of T cells. Accordingly, in some experiments in which α-lactose was added during the activation of CD8^+^ T cells their activation was impaired, which might suggest the role of other players (Figure S4E). Therefore, the experiments aimed at the neutralization of galectin-3 function by an antibody provided more relevant information with respect to its intracellular versus extracellular role in cellular activation (Figures 6A–6C). That the antibody used could affect extracellular galectin-3 neutralization was shown earlier (Gordon-Alonso et al., 2017, Yip et al., 2017). Our experiments also demonstrate the binding of the antibody to extracellular galectin-3 (Figures S4F and S4G).

We observed that upon their co-culture with cognate-peptide-pulsed APCs, galectin-3 rapidly recruited to the immunological synapse in CD8^+^ T cells undergoing a primary as well as a secondary stimulation (Figures 3, 4, and 5). Expression of galectin-3 compromised the magnitude of virus-specific CD8^+^ T cells to clear the virus, as galectin-3-deficient CD8^+^ T cells showed an enhanced frequency for almost all epitopes of MHV68. The ability of galectin-3 to constrain anti-viral immunity is counterintuitive, but its presence at the immunological synapse could help attenuate spontaneous activation of the TCR to avoid immunopathological reactions, particularly to self-antigens (Radosavljevic et al., 2012). Development and maintenance of peripheral CD8^+^ T cells requires sustained low-affinity interactions that involve self-peptides loaded onto MHC I molecules (Ziegler et al., 2009). Therefore, the presence of galectin-3 at synapse could act as a break to constrain any possible hyperactivity. Galectin-3 is also thought to compromise the activity of cytotoxic CD8^+^ T cells during some autoimmune diseases as well as in the tumor microenvironment (Fortuna-Costa et al., 2014, Kouo et al., 2015, Nangia-Makker et al., 2008). In a tumor environment the interaction of galectin-3 with T cells effector cytokine IFN-γ compromised its protective function, and the effect is extracellular in nature. However, how galectin-3 acts to constrain anti-viral CD8^+^ T cell immunity during HV infection is still not understood and was elucidated in this study. We found that galectin-3 contributed by cells other than CD8^+^ T cells had little influence in impairing the induction, activation, and expansion of MHV68- specific TN CD8^+^ T cells *in vivo* (Figure 9). The role of galectin-3 in CD8^+^ T cells during a γ-HV infection is thus cell autonomous.

The distribution of single-positive CD4^+^ and CD8^+^ T cells in the thymus of galectin-3 KO and WT animals were similar, suggesting that during development of these cells galectin-3 may not be playing a major role and predominantly regulates reactivity of T cells in the periphery (Figure S7). The observation that memory CD8^+^ T cells when recalled by antigenic stimulation does induce the recruitment of regulatory galectin-3 to immune synapse can have implication in designing the regimen for priming and boosting vaccine-specific CD8^+^ T cell immunity. Our results suggest that modifying galectin-3 function in CD8^+^ T cells and not in a generic manner could be considered as a strategy to enhance anti-viral CD8^+^ T cell immunity, for example, through the disruption of its localization to the immune synapse by using small molecules or intrabodies, by manipulation of its glycosylation status, or even by adjusting the expression levels of galectin-3 itself.

### Limitation of Study

The aim of this study was to investigate the role of galectin-3 in influencing anti-γ-HV immunity; an extensive kinetics of T cells activation and differentiation in an environment lacking in galectin-3 versus the one sufficient for its response was not investigated and constitutes part of our ongoing investigations.

## Methods

All methods can be found in the accompanying Transparent Methods supplemental file.

## Supplemental Information

Document S1. Transparent Methods, Figures S1–S7, and Tables S4 and S5

Table S1 Differential Transcriptome of Naive and MHV68-Expanded CD8^+^ TN Cells, Related to Figures 1 and 2

Table S2 Biological Processes That Are Affected by Upregulated Transcripts in Activated CD8^+^ TN cells (as Analyzed by DAVID Gene Ontogeny Biological Processes; Percentage of Enrichment of Genes >1.0% and p Value > 0.05), Related to Figures 1 and 2

Table S3 Biological Processes That Are Affected by Downregulated Transcripts in Activated CD8^+^ TN Cells (as Analyzed by DAVID Gene Ontogeny Biological Processes), Related to Figures 1 and S1
