# Supplementary material for: Galectin-3 Regulates γ-Herpesvirus Specific CD8 T Cell Immunity
Source: iScience. 2018 Oct 17;9:101–19. doi: 10.1016/j.isci.2018.10.013 (PMC6214866; doi:10.1016/j.isci.2018.10.013)
Supplement: Document S1. Transparent Methods, Figures S1–S7, and Tables S4 — and S5 [file mmc1.pdf]

ISCI, Volume *9*

Supplemental Information

Galectin-3 Regulates γ-Herpesvirus Specific CD8 T Cell Immunity

Manpreet Kaur, Dhaneshwar Kumar, Vincent Butty, Sudhakar Singh, Alexandre Esteban, Gerald R. Fink, Hidde L. Ploegh, and Sharvan Sehrawat

Figure S1

A

#Sample	UniqPer10M	Total	UniquelyMapping	%UniqMapping	RiboMapping	%Ribo	SpliceJunctions	%Jxns	Exon/Intron	Exon/Intergenic
Naive	0.62	98405474	74296323	75.5	475650	0.64	7411715	9.98	80.72	1859.99
Day6	0.59	107360448	82907794	77.22	986561	1.19	10282808	12.4	198.8	4125.19

#Sample	log2(m(3'/5'))	log2(m(3'/CDS))	log2(m(CDS/5'))	sd(3'/5')	sd(3'/CDS)	sd(CDS/5')	Insert size Mean (bp)	Insert size Stdv (bp)
Naive	0.38	-0.21	0.59	1.43	0.97	1.33	173.6	67.1
Day6	0.31	-0.25	0.55	1.53	1.1	1.45	179	60.3

B

Gene	Naïve RPKM	D6 RPKM	Day6/Naive
Tap1	153	147	1.041
Beta2m	2362	1743	1.3
H2(D1)	156	169	0.98

C

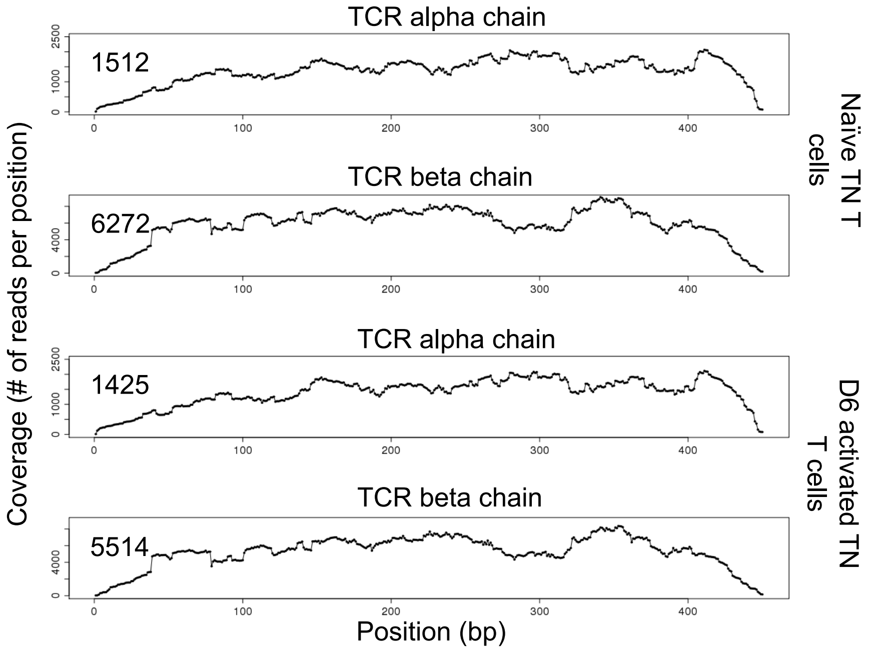

D

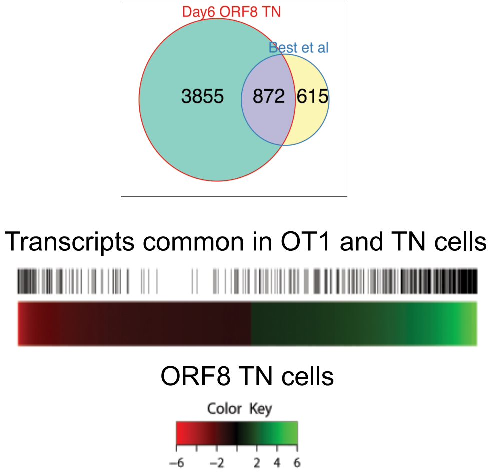

E

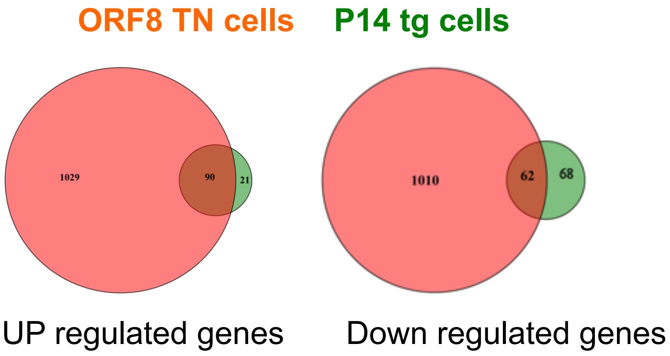

Figure S1 Analysis of RNA seq data obtained from H-2K^{b}-ORF8 TCR TN naïve and activated CD8^{+} T cells to determine its quality. (Related to figure 1& 2)

A. Key attributes for analyzing the quality of RNA seq data. B. The number of reads (RPKM) recorded for some of the control genes. RPKM (Reads per kilobase per million mapped reads) = total exon reads/mapped reads (million) x exon length (Kb) C. Intensity of the reads recovered from RNAseq data present in the rearranged VDJ and VJ of β- and α-chains of TCR in naïve and activated H-2K^{b}-ORF8 TCR TN CD8^{+} T cells. D-E. A comparison of transcriptome of TN cells CD8^{+} T cells, Tg OT1 cells and Tg P14 CD8^{+} T cells that were activated by γ-herpesvirus, Listeria monocytogenes and Lymphocytic choriomeningitis virus (LCMV) Armstrong infection respectively. D. Upper panel; Venn diagram shows the comparisons of transcriptome of OT1 cells and ORF8 TCR TN CD8^{+} T cells that were differentially expressed atleast 1.5 fold in both the cell types. Lower panel; Heat map of 1.5 fold differentially expressed transcripts in ORF8 TCR TN CD8^{+} T cells (lower heat map) and those that are common in OT1 cells and ORF8 TN cells (upper heat map). E. A comparison of differential transcriptome of ORF8 TN cells and P14 tg cells in the acute phase of a MHV68 and LCMV infection. Pi chart shows the numbers of genes up or down regulated by two fold in the respective cell types.

Figure S2

A

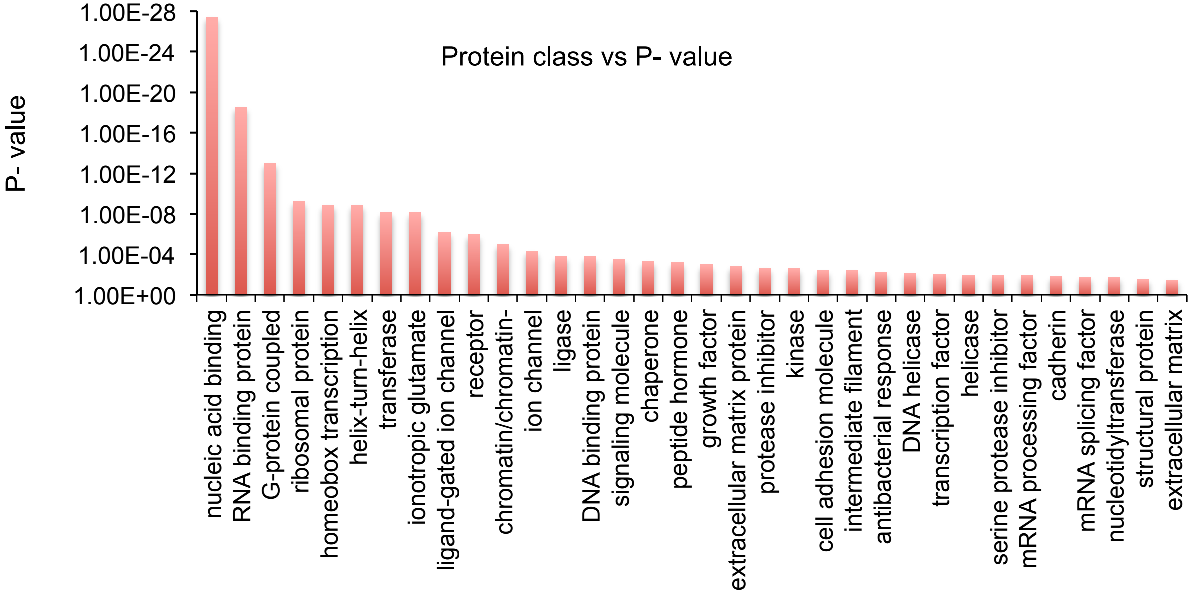

B

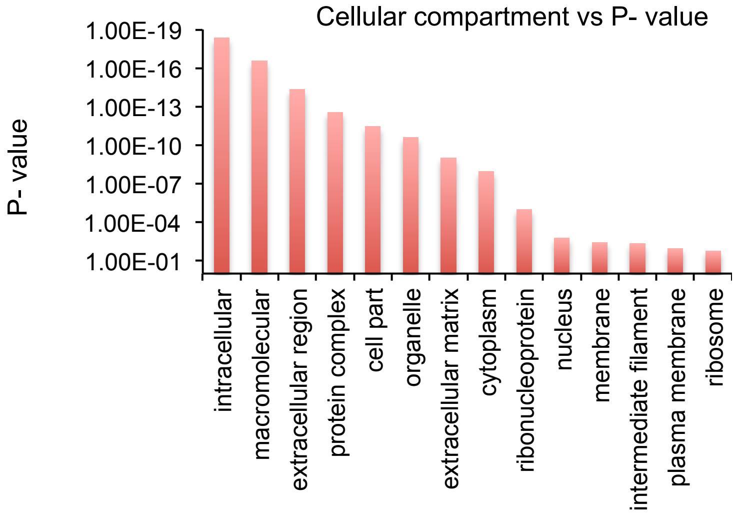

Figure S2 Gene ontology (GO) panther pathway analysis of differentially expressed transcripts in activated and naïve ORF8 TCR TN CD8^{+} T cells. (Related to figure 1)

A. Bar diagram depicts the p-values of transcripts represented for the class of proteins. B. Bar diagram depicts the p-values of transcripts represented for cellular compartment.

Figure S3

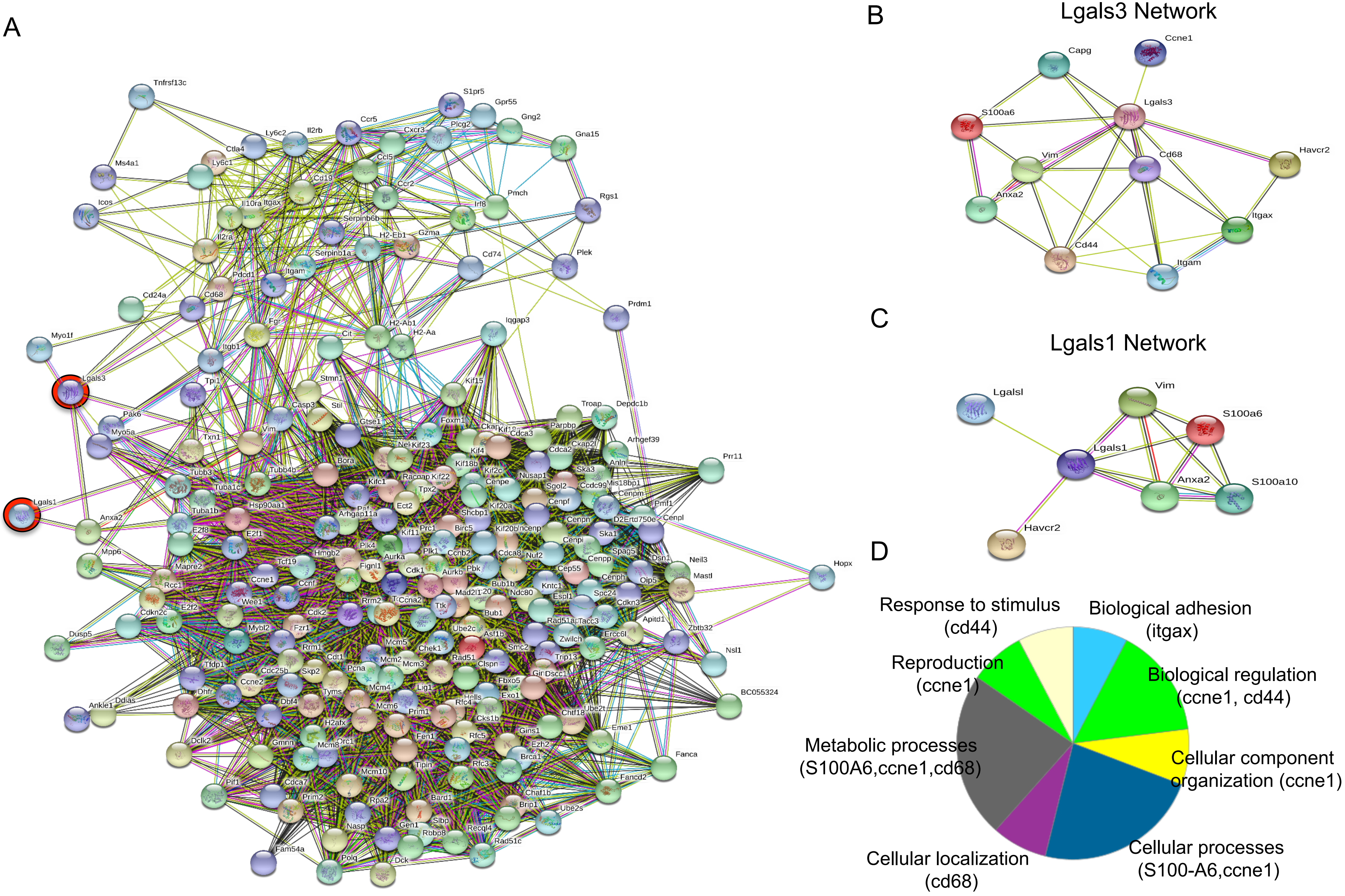
A
B
Lgals3 Network
Capg
Ccne1
Lgals3
S100a6
Havcr2
Vim
Cd68
Anxa2
Itgax
Cd44
Itgam
C
Lgals1 Network
Lgalsl
Vim
S100a6
Lgals1
Anxa2
S100a10
Havcr2
D
Response to stimulus (cd44)
Biological adhesion (itgax)
Reproduction (ccne1)
Biological regulation (ccne1, cd44)
Metabolic processes (S100A6,ccne1,cd68)
Cellular component organization (ccne1)
Cellular localization (cd68)
Cellular processes (S100-A6,ccne1)

Figure S3 Computational analysis of RNAseq data to generate STRING network to investigate protein-protein interactions (PPI). (Related to figure 2)

A. STRING network of differentially expressed genes having a cut off criteria of 5RPKM in either samples and a two fold change in expression between naïve and activated TN cells. Hub genes having equal to or more than 5 interactions with a p value <1.0e-16 were used for network generation. Members of galectin family are highlighted with red circles. B and C. STRING Network is generated to show Lgals genes as hub genes, Lgals3 (B) and Lgals1(C). Lgals3 interacted with 10 partners while Lgals1 interacted with 6 partners. Both networks had a PPI enrichment p value < 1.0e-16. D. Lgals3 gene and its interacting partner (7 out of 10 genes were mapped through PANTHER) to investigate different biological process. *Itgam, capg*, and *Anxa2* were not mapped through PANTHER.

Figure S4

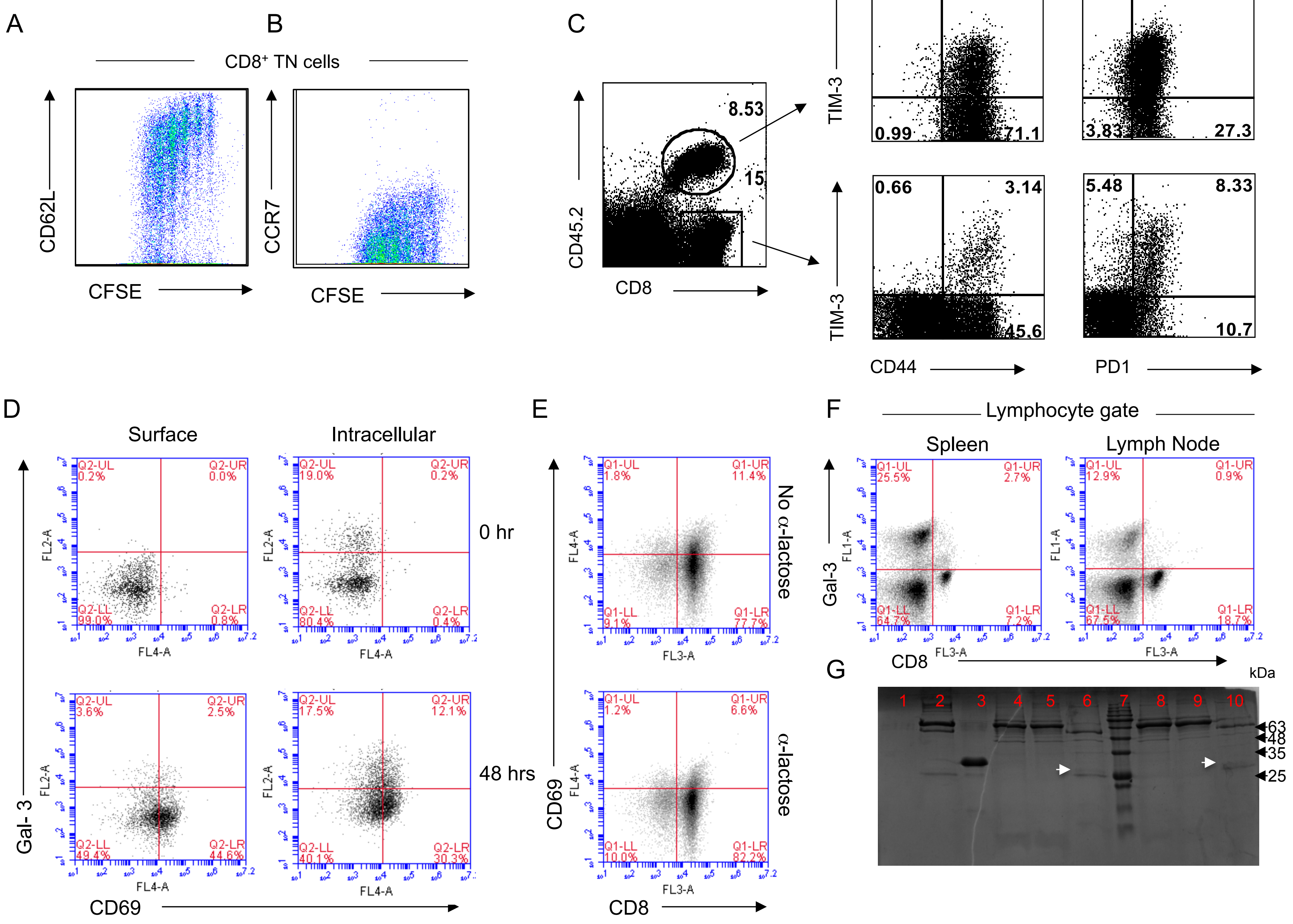
A
B
CD8+ TN cells
CD62L
CFSE
CCR7
CFSE
C
CD45.2
CD8
8.53
15
TIM-3
0.85
27.1
0.99
71.1
6.32
62.6
3.83
27.3
0.66
3.14
45.6
5.48
8.33
10.7
CD44
PD1
D
Surface
Intracellular
Q2-UL 0.2%
Q2-UR 0.0%
Q2-LL 99.0%
Q2-LR 0.8%
Q2-UL 19.0%
Q2-UR 0.2%
Q2-LL 80.4%
Q2-LR 0.4%
0 hr
Q2-UL 3.6%
Q2-UR 2.5%
Q2-LL 49.4%
Q2-LR 44.6%
Q2-UL 17.5%
Q2-UR 12.1%
Q2-LL 40.1%
Q2-LR 30.3%
48 hrs
FL2-A
FL4-A
Gal- 3
CD69
E
Q1-UL 1.8%
Q1-UR 11.4%
Q1-LL 9.1%
Q1-LR 77.7%
No α-lactose
Q1-UL 1.2%
Q1-UR 6.6%
Q1-LL 10.0%
Q1-LR 82.2%
α-lactose
FL4-A
FL3-A
CD69
CD8
F
Lymphocyte gate
Spleen
Lymph Node
Q1-UL 25.5%
Q1-UR 2.7%
Q1-LL 64.7%
Q1-LR 7.2%
Q1-UL 12.9%
Q1-UR 0.9%
Q1-LL 67.5%
Q1-LR 18.7%
Gal-3
FL1-A
FL3-A
CD8
G
kDa
1 2 3 4 5 6 7 8 9 10
63
48
35
25

Fig S4 Analyzing the expression of some of the gene at protein level in activated TN cells as compared to naïve TN cells. (Related to figure 1 &2)

A and B. CFSE labeled ORF8 TN T cells were stimulated with BMDCs pulsed with the cognate KNYIFEEKL peptide for three days. Representative FACS plots show the expression of CD62L (A) and CCR7 (B) in dividing TCR TN $CD8^+$ T cells. Representative FACS plots are shown. C. Representative FACS plots show the expression of TIM-3 and PD1 by donor and endogenous $CD8^+$ T cells. For each experiment at least four animals were included and experiments were repeated two times with similar results. D. *In vitro* activation of magnetically sorted $CD8^+$ T cells using anti-CD3 (coated) and CD28 (soluble) antibodies to measure intracellular (permeabilized cells) and surface (without permeabilization) galectin-3. The cells were stimulated for two days and the expression level of galectin-3 were measured as described in transparent methods section. Activated cells predominantly up regulated intracellular galectin-3 but its expression on cell surface was affected to a lesser extent. E. Measuring the influence of a-lactose on $CD8^+$ T cell activation. $CD8^+$ T cells were purified from lymph node of C57BL/6 mouse and incubated with 277mM of α-lactose for 1 hour at 37°C. The cells were then washed three times with PBS and stimulated with anti-CD3 (coated) and anti-CD28 (soluble) for 12 hrs at 37°C. After activation the cells were washed and stained with anti-CD69 and anti-CD8 antibodies. F-G. A demonstration that the clone (B2C10) of antibody used for neutralization of galectin-3 can bind and detect extracellular surface expressed galectin-3. F. Splenocytes from C57BL/6 mice were isolated to stain for CD8 and galectin-3. Gated lymphocytes are shown for anti-CD8 and galectin-3 stainings. While $CD8^+$ T cells expressed low levels of galectin-3, $CD8^-$ T cells demonstrate significant population of surface galectin-3 expressing cells. G. A galectin-3 pull-down by the neutralizing antibody (B2C10) from the lysates of spleen cells and lymph node cells. Different samples prepared from pull down experiments were resolved on a 12% SDS-PAGE. lane 1: dye, lane 2: beads with IgG1 (blocking antibody, 3μg), lane 3: recombinant galectin 3 (3μg), lane 4: input splenocytes lysate, lane 5: residual sample from splenocytes lysate after pull-down, lane 6; pull-down fraction from splenocytes lysate, lane 7: molecular weight marker (MWM), lane 8: input lymph node cell lysate, lane 9: residual sample from splenocytes lysate after pull-down, lane 10: pull-down fraction from lymph node cells. In lanes 6 and 10, a faint band of galectin-3 corresponding to 30kDa is retrieved (white arrow marked), bonds corresponding to heavy chain (~50kDa) and light chain (25kDa) of IgG1 molecules are also visible. High molecular mass bands might represent aggregated products and the carrier protein in antibody preparation.

Figure S5

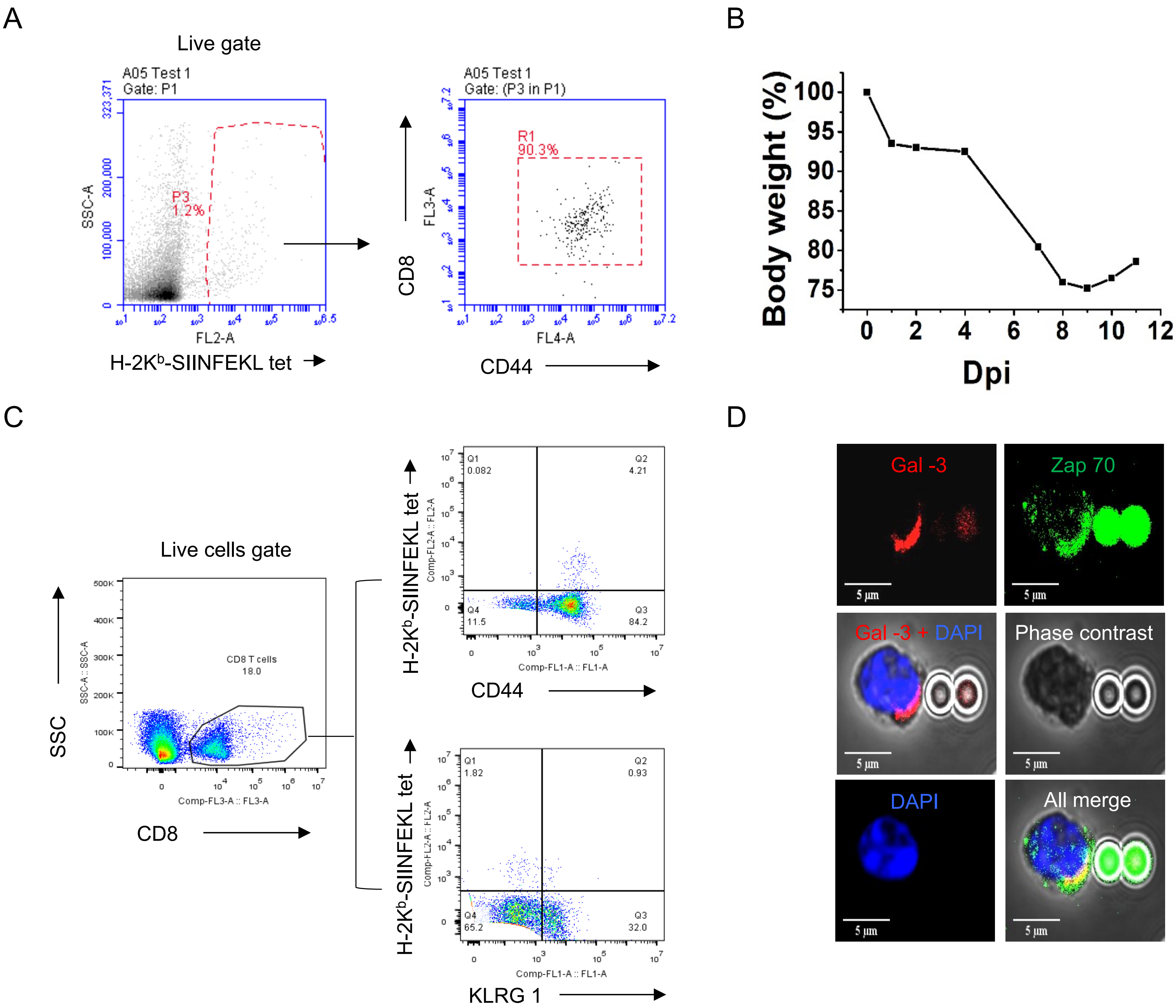
A
Live gate
A05 Test 1
Gate: P1
P3
1.2%
SSC-A
FL2-A
H-2Kb-SIINFEKL tet
A05 Test 1
Gate: (P3 in P1)
R1
90.3%
FL3-A
CD8
FL4-A
CD44
B
Body weight (%)
Dpi
C
Live cells gate
CD8 T cells
18.0
SSC
CD8
Q1
0.082
Q2
4.21
Q3
84.2
Q4
11.5
H-2Kb-SIINFEKL tet
CD44
Q1
1.82
Q2
0.93
Q3
32.0
Q4
65.2
KLRG 1
D
Gal -3
Zap 70
Gal -3 + DAPI
Phase contrast
DAPI
All merge
5 µm

Figure S5 Phenotypic analysis of antigen-specific CD8$^+$ T cells obtained from mice infected with MHV68 M2-SIINFEKL and WSN-SIINFEKL. (Related to figure 5)

A. 50x10^3 OT1 cells were transferred into C57BL/6 mice, which were then infected with MHV68 M2-SIINFEKL through intranasal route. On 5dpi, PBMCs were analyzed for SIINFEKL specific CD8$^+$ T cells. Representative FACS plots are shown. B-D. C57BL/6 mice were infected with WSN-SIINFEKL through intranasal route and disease progression and phenotypic analysis of antigen-specific CD8$^+$ T cells was measured. B. Body weight of infected animals was monitored for up to 12 days and is shown as change in body weight as compared to the original. C. On 6dpi, mediastinal LN single cell suspensions were analyzed for the phenotype of endogenous SIINFEKL specific CD8$^+$ T cells. Representative FACS plots are shown. D. H-2K^b-SIINFEKL-specific CD8$^+$ T cells were magnetically sorted and analyzed for co-localization of galectin-3 and Zap70 towards the immune synapse. Representative confocal images are shown.

Figure S6

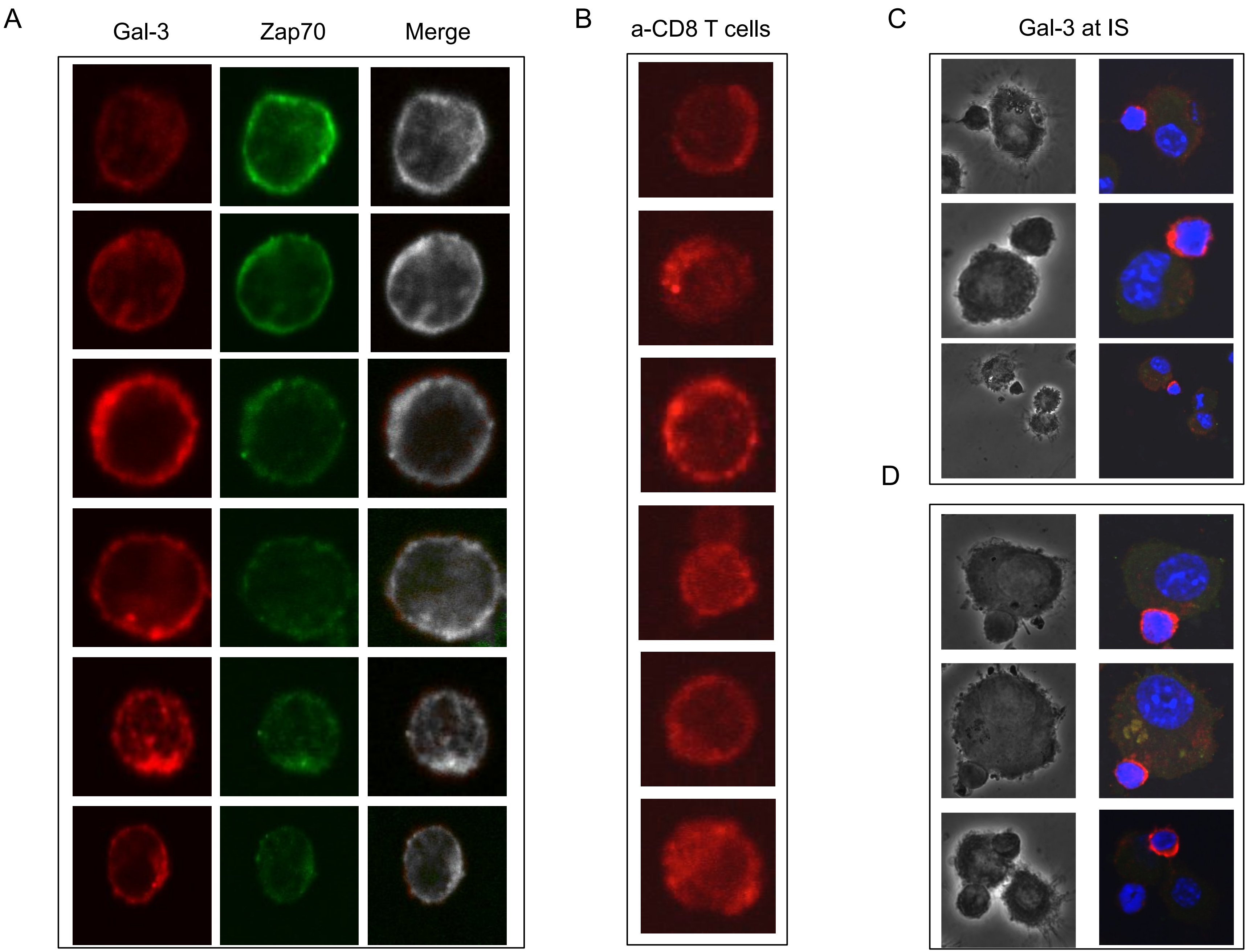

Figure S6 Additional images to demonstrate the recruitment of galectin-3 at immunological synapse. (Related to figure 4)

A. Previously activated OT1 CD8$^+$ T cells during infection of naïve OT1 recipient mice with MHV68-M2-SIINFEKL were isolated during the acute phase of response at 6dpi and analyzed for co-localization of galectin-3 and Zap70. Six representative images are shown. B. Representative images of sorted OT1 cells incubated in the absence of peptide pulsed APCs. C-D. Representative images to demonstrate recruitment of OT1 cells' expressed galectin-3 at immunological synapse upon incubation with SIINFEKL pulsed APCs at 10min (C) and 1hr (D).

Figure S7

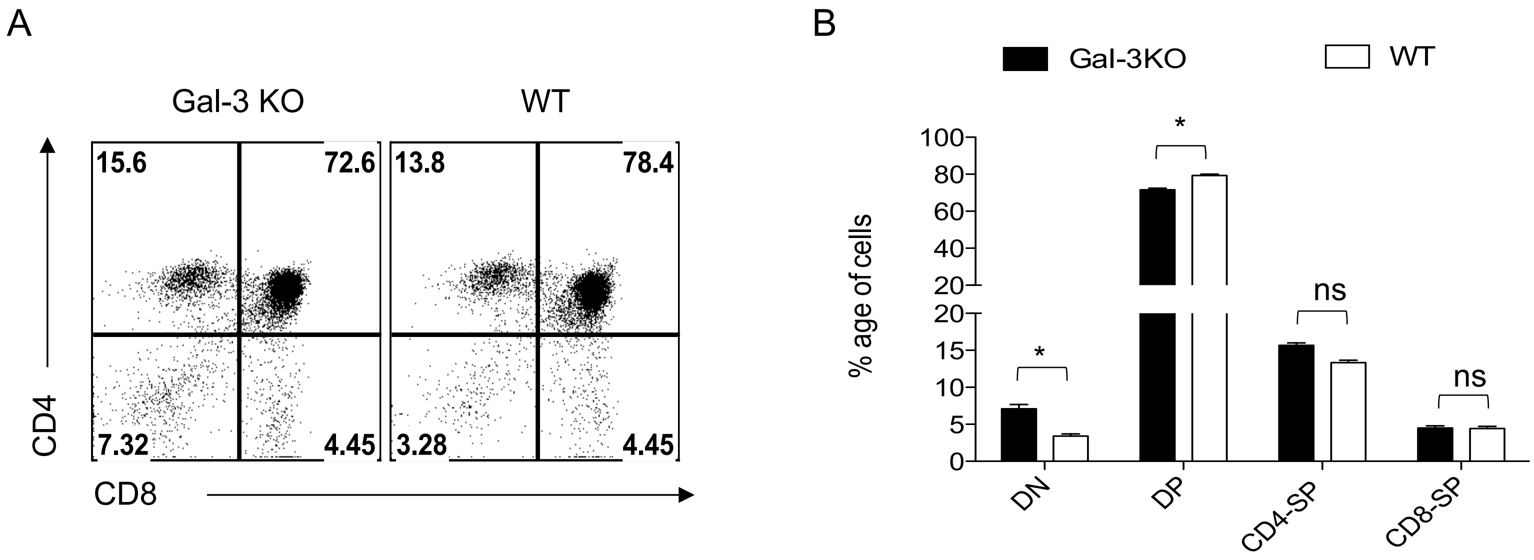

Figure S7 Comparison of thymic cellular distribution in galectin-3 KO and WT animals. (Related to figure 6 to 9)

A. FACS plots show the frequencies of single positive, double positive and double negative for $CD4^+$ and $CD8^+$ T cells. B. Bar diagram represent the frequencies of indicated cell populations from four animals in each group.

Table S4: A list on unmapped genes using Gene Ontology analysis (Related to figure 1)

0610007P14Rik	1810043G02Rik	4632434I11Rik	AI467606	BC094916	D14Abb1e	Gm9846	Mll5	Phf17	Wdr20a	Zfp526
0610009D07Rik	2010012O05Rik	4833420G17Rik	AI597468	Beta-s	D4Wsu53e	Gpi1	Mnf1	Pion	Wdr67	Zfp579
0610010F05Rik	2010015L04Rik	4930422G04Rik	AI837181	Bloc1s2a	D5Ertd579e	Grlf1	Mrp63	Rab1	Wdr85	Zfp580
0610010K14Rik	2010107E04Rik	4930427A07Rik	AI846148	Bmyc	D8Ertd82e	Gtpbp5	Nrp	Rnaset2a	Zfp110	Zfp592
0610030E20Rik	2010111I01Rik	4930432K21Rik	AK010878	C030006K11Rik	Ddx39	Gyg	Nt5c3l	Rnaset2b	Zfp12	Zfp652
0610037L13Rik	2210016L21Rik	4930444A02Rik	Akr1b3	C030046E11Rik	Dnahc8	Gyk	Nup62-il4i1	Rpl34-ps1	Zfp146	Zfp653
1110004F10Rik	2210404J11Rik	4930453N24Rik	Akr1e1	C230052I12Rik	Dnalc4	H2-Ke2	Pagr1a	Rpl36al	Zfp182	Zfp661
1110008F13Rik	2210404O07Rik	4930486L24Rik	Anks1	C330006A16Rik	Dnase2a	H2-Ke6	Pcdhga1	Rps3a1	Zfp207	Zfp664
1110008J03Rik	2300009A05Rik	4930503L19Rik	Atad3a	C330027C09Rik	E130309D02Rik	H2-T9	Pcdhga10	Serpinb6a	Zfp260	Zfp672
1110008P14Rik	2310011J03Rik	4930579G24Rik	Atp5k	C87436	E130311K13Rik	H3f3b	Pcdhga11	Sfrs18	Zfp276	Zfp687
1110034G24Rik	2310022B05Rik	4932438A13Rik	Atpbd4	C920025E04Rik	E330009J07Rik	Hba-a1	Pcdhga12	Solh	Zfp280c	Zfp692
1110059E24Rik	2310035C23Rik	4933434E20Rik	AU022252	Calm2	E430025E21Rik	Hba-a2	Pcdhga2	Spata5l1	Zfp280d	Zfp71-rs1
1190002N15Rik	2310039H08Rik	5730508B09Rik	AW549877	Calm3	Eef1b2	Hist1h4c	Pcdhga3	Spin2	Zfp281	Zfp710
1190007I07Rik	2310042D19Rik	5830415F09Rik	AW554918	Car12	Efha2	Hist1h4i	Pcdhga5	Stx4a	Zfp287	Zfp74
1300018J18Rik	2410066E13Rik	6030458C11Rik	B630005N14Rik	Car15	Eif2c1	Hmga1-rs1	Pcdhga6	Stxbp3a	Zfp318	Zfp740
1500009L16Rik	2410131K14Rik	6330403M23Rik	B930041F14Rik	Car2	Eif2c2	Hnrpll	Pcdhga7	Supt16	Zfp329	Zfp770
1500032L24Rik	2510002D24Rik	6330416G13Rik	Baat1	Car5b	Eif2c3	Hras1	Pcdhga8	Supt20	Zfp335	Zfp771
1600002H07Rik	2610019F03Rik	8430410A17Rik	BC003965	Ccdc90a	Fam108c	Jhdm1d	Pcdhga9	Supt4a	Zfp354c	Zfp784
1600014C10Rik	2610301B20Rik	9030617O03Rik	BC004004	Cd24a	Fam86	Klhdc5	Pcdhgb1	Sypl	Zfp365	Zfp821
1700017B05Rik	2700029M09Rik	9430023L20Rik	BC016423	Cenpc1	Fam92a	Klra12	Pcdhgb2	Tgtp2	Zfp367	Zfp827
1700020L24Rik	2700060E02Rik	9930012K11Rik	BC029214	Chmp1b	Fasl	Klra23	Pcdhgb4	Tmem170	Zfp369	Zfp85-rs1
1700025G04Rik	2700094K13Rik	9930104L06Rik	BC030336	Cldn25	Fem1a	Lrdd	Pcdhgb5	Tmem48	Zfp382	Zfp865
1700037H04Rik	2810403A07Rik	A130010J15Rik	BC030867	Cnih	Fh1	Lrrc33	Pcdhgb7	Tmem8	Zfp422	Zfp932
1700066M21Rik	2810417H13Rik	A230046K03Rik	BC037034	Csda	Gbp3	Lsmd1	Pcdhgb8	Tmsb15b2	Zfp445	Zkscan14
1700088E04Rik	3010026O09Rik	A230050P20Rik	BC048403	Ctps	Gm13826	Man1a	Pcdhgc3	Trp53	Zfp451	Zkscan6
1700094D03Rik	3110057O12Rik	A430005L14Rik	BC049715	Cxx1b	Gm20604	Mettl21d	Pcdhgc4	Trp53bp2	Zfp507	Znrd1as
1810013L24Rik	3230401D17Rik	A730008H23Rik	BC052040	Cyp4v3	Gm5506	Mll1	Pcdhgc5	Trp53i13	Zfp513	
1810022K09Rik	4632415K11Rik	AA414768	BC055324	Cyp51	Gm5918	Mll2	Pgm1	Txn1	Zfp518a	
1810037I17Rik	4632428N05Rik	AB124611	BC056474	D030056L22Rik	Gm6251	Mll3	Phf15	Ube2cbp	Zfp523	

Table S5 -Hub genes and their numbers of interactions (Cut Off ≥5 interactions) (Related to figure 2 & 3)

Genes	Interaction	Genes	Interaction	Genes	Interaction	Genes	Interaction
Mcm5	141	Cdkn3	55	Rfc4	27	Cdc25b	11
Prc1	136	Ckap2	55	Kif15	26	Fanca	11
Mad2l1	129	Ccne1	54	Tyms	26	Zbtb32	11
Kif20a	125	Fzr1	54	Racgap1	26	Ube2s	11
Rad51ap1	119	Ercc6l	54	Rad51c	26	Tpi1	11
Top2a	118	Fignl1	54	Tuba1b	25	Pak6	11
Smc2	117	Kif22	53	Nasp	24	Bard1	10
Zwilch	116	Kntc1	52	Dscc1	24	Plcg2	10
Cenpf	114	Rpa2	52	Fancd2	23	Cd24a	10
Kifc1	111	Rfc3	51	Tubb3	23	**Lgals3**	**10**
Cdca8	109	Gins2	51	Myo5a	22	Il10ra	10
Ect2	107	Kif23	51	Pbk	21	Hsp90aa1	9
Lig1	105	Tipin	51	Tuba1c	21	Cit	9
Cep55	103	Ccne1	51	Wee1	20	Ly6c2	9
Birc5	102	Anln	50	E2f2	20	Il2ra	9
Kif20b	101	Ccna2	50	Tubb4b	20	Cdkn2c	9
D2Ertd750e	101	Bub1	50	Pmf1	20	H2-Aa	8
Ube2c	100	Ezh2	50	H2-Ab1	20	Fbxo5	8
Ttk	98	Gen1	49	Cdca3	20	Kif11	8
Cdca2	97	H2afx	48	Polq	20	S1pr5	8
Rfc5	92	Rrm1	48	Shcbp1	20	Tnfrsf13c	8
Tacc3	92	Cenpn	47	Cd68	20	Vim	8
Oip5	90	Arhgef39	46	Mcm4	19	Dck	8
Mcm3	89	Mcm8	46	Stil	18	Ly6c1	8
Kif2c	89	Ccne2	45	E2f8	18	Irf8	8
Mcm2	88	Pcna	45	Prr11	18	Gng2	7
Spc24	88	Rad51	45	Mcm10	17	Anxa2	7
Cenpi	88	Cdca7	45	Il2rb	17	Prdm1	7
Arhgap11a	88	Parpbp	44	Itgb1	17	Plek	7
Espl1	86	Tpx2	43	Ska3	17	Mapre2	7
Cenph	86	Depdc1b	42	Trip13	16	Ankle1	7
Ccnf	83	Cks1b	40	Fam54a	16	Nsl1	6
Nusap1	81	E2f1	39	Cdk1	15	Gna15	6
Cenpe	79	Gins1	39	Rbbp8	15	**Lgals1**	**6**
Kif4	75	Neil3	38	Apitd1	15	Dhfr	6
Ccdc99	75	Slbp	37	Bora	15	Mpp6	6
Bub1b	74	Tfdp1	35	Pif1	15	Cd74	5
Orc1	74	Cenpl	34	Itgax	15	Gmnn	5
Clspn	74	Nuf2	34	Serpinb1a	15	Cdt1	5
Dbf4	73	Eme1	34	Serpinb6b	15	H2-Eb1	5
Hmgb2	69	Itgam	34	Mastl	15	Cdc20	5
Exo1	67	Troap	33	Aurkb	14	Asf1b	5
Cenpm	67	Casp3	33	Ccr2	14	Txn1	5
Tcf19	67	Plk4	32	Cxcr3	14	Pmch	5
Plk1	64	Mcm6	32	Chaf1b	14	Gpr55	5
Ccnb2	64	Prim1	31	Kif18b	14	Gzma	5
Skp2	64	Recql4	31	Ccl5	14	Mybl2	5
Chek1	62	Fen1	31	Prim2	13	Brca1	5
Foxm1	61	Hells	30	Rrm2	13	Pdcd1	5
Spag5	61	Ndc80	29	Cenpp	13	Ctla4	5
Ska1	60	Ccr5	29	Dclk2	13	Icos	5
Gtse1	60	Kif18a	28	BC055324	13	Rgs1	5
Ckap2l	60	Incenp	28	Iqgap3	13	Hopx	5
Chtf18	59	Cdk2	28	Cd19	12	Dusp5	5
Paf	57	Sgol2	28	Ms4a1	12	Myo1f	5
Mis18bp1	56	Brip1	28	Stmn1	12	Ddias	5
Aurka	55	Ube2t	27	Rcc1	12		
Dsn1	55	Nek2	27	Fgr	12		

Transparent Methods

Mice, virus and cell lines used

Murine γ-herpesvirus (MHV68)-specific $CD8^+$ T cell transnuclear (TN) mice were generated and bred onto a C57BL/6 $Rag1^{-/-}$ background (Sehrawat et al., 2012). TCR TN mice, C57BL/6 mice WT mice, OT1 TCR transgenic (tg) mice and galectin-3 knock out (KO) mice were bred and maintained at the AALAC-accredited animal facility of the Whitehead Institute for Biomedical Research, Cambridge, MA or at the Small Animal Facility for Experimentation at the Indian Institute of Science Education and Research Mohali. Age and gender-matched mice were used for experiments. All studies were carried out in accordance with procedures approved by the Massachusetts Institute of Technology Committee on Animal Care (CAC protocol # 1011-123-14) and Institute Animals Care and Use Committee (IACUC) of IISER Mohali. Whitehead Institute's Animal Welfare Assurance was approved and the approval number is 11/3/2009 (IACUC, A3125-01). Institutional Animal Ethics Committee (IAEC) of Indian Institute of Science Education and Research Mohali (IISER Mohali), constituted by the Committee for the Purpose of Control and Supervision of Experiments on Animals (CPCSEA) which is established under Chapter 4, Section 15(1) of the Prevention of Cruelty to Animals Act 1960. IAEC approved all the protocols and their numbers are; (IISERM/SAFE/PRT/2016/008, 009 and IISERM/SAFE/PRT/2017/011). All the experiments were performed strictly in accordance with the approved protocols. The cell lines (Vero cells, 3T12-3 and MDCK were obtained from ATCC. All the viruses used were described earlier (Sehrawat et al., 2013). The MHV-68, its recombinant version encoding for a SIINFEKL peptide (MHV68-M2 SIINFEKL) and influenza virus encoding SIINFEKL (WSN-SIINFEKL) were grown in 3T12-3 and MDCK cells respectively and stored at −80°C until further use.

Antibodies and Other Reagents

Fluorochrome-conjugated antibodies against mouse CD16/32, CD3ε, CD8a, CD4, CD62L, CD69, CD44, B220, IFN-γ, CD11c, CD11b, CD45.1, CD45.2, PD1, KLRG1, TIM-3 and CCR7 were purchased from BD PharMingen. Anti-galectin-3 alexafluor 647 and anti-galectin-3 PE antibody were obtained from eBioscience. Anti-Zap70-FITC was obtained from BD biolegend. A neutralizing anti-galectin-3 antibody (clone B2C10) was obtained from Thermofisher Scientific. Peptides were either synthesized in house at the Massachusetts Institute of Technology biopolymer facility or were obtained from GL Biochem. H-2K^b tetramers loaded with the indicated peptides were generated and used essentially as described (Altman et al., 1996). Tetramers carrying different peptides were generated by photochemical exchange of a conditional ligand (Bakker et al., 2008). The peptide used for tetramer generation and intracellular cytokine staining (ICCS) assays include those derived from ORF9 (SVYGFTGV; H-2K^b restricted) and ORF75c (KSLTYYKL; H-2K^b restricted, SAIENYETF; H-2D^b restricted) for early antigens and ORF6 (AGYIYYQL; H-2K^b restricted, AGPHNDMEI; H-2D^b restricted), ORF8 (KNYIFEEKL; H-2K^b restricted), ORF17 (SAITNHAAF; H-2D^b restricted) and ORF61 (p79) (TSINFVKI; H-2K^b restricted). For some experiments chicken ovalbumin-derived SIINFEKL peptide was used to probe OT1 or the endogenous cells.

Isolation of CD8$^+$ T cells

In order to isolate minimally manipulated CD8$^+$ T cells, negative selection kits from Miltenyi Biotec, Stem cell technology or Dynabeads™ Mouse CD8$^+$ T cells isolation kits were used. The purity of the CD8$^+$ T cells thus obtained was ascertained by flow cytometry. All procedures for cell separation were performed as per manufacturer's instructions. For some experiments cell sorting of fluorescent antibody-labeled cells was performed using a FACSaria II or FACSfusion instruments. The desired cell populations were obtained via a “dump” gate using antibodies against markers specific for cells other than CD8$^+$ T cells. For some experiments, antigen-specific

$CD8^+$ T cells were isolated from infected mice using magnetic beads coated with class I MHC monomers.

Adoptive transfer of cells and virus infection

$50x10^3$ negatively separated MHV68-gB-ORF8-TCR TN cells from H-$2K^b$ -MHV68-gB-ORF8-TCR TN mice were adoptively transferred intravenously into CD45.1 congenic C57BL/6 or WT C57BL/6 mice one day before intra peritoneal infection with $5x10^5$ pfu of MHV68. In some experiments OT1 cells obtained from OT1 TCR tg mice were transferred in WT C57BL/6 mice, which were then infected with $5x10^5$ pfu of MHV68 M2-SIINFEKL. In order to measure responses of endogenous memory $CD8^+$ T cells, an intranasal infection of 10 weeks old mice with WSN-SIINFEKL ($5x10^2$ pfu) was performed. The body weight was measured until 12 days post infection (dpi). Infected animals were euthanized using CO_2 asphyxiation at different dpi for further analyses. Single cell suspensions obtained from lymphoid organs were analyzed for the number and phenotype of expanded cells. WT and Gal-3 KO mice were infected with MHV68 by intraperitoneal or intranasal route. Lymphoid organs were analyzed to measure $CD8^+$ T cell responses. Lung tissues were collected to estimate viral loads.

RNA sequence analysis

FACS-purified naïve and MHV68 expanded K^b-ORF8-TCR TN $Rag1^{-/-}$ $CD8^+$ T cells were used for RNA isolation. The purity of sorted cells was verified by flow cytometry and was routinely > 99 percent. RNA was isolated from sorted cells using a kit from Qiagen. A Trueseq RNA sample Prep kit v2 from Illumina was used for RNA processing. 1µg of input total RNA was used and 18 cycles of PCR were performed and libraries were constructed. Paired end sequencing was performed. The sequence was mapped to mm9 using ELAND tool. Reads were counted in exons REFSEQ transcripts. Then, the count was normalized for reads per kb of mRNA per million (RPKM). For most analyses, transcripts below 5RPKM in paired samples

were eliminated and genes that were selected had a more than 1.5 fold or more than two-fold difference in any comparison as indicated in figure legends.

Network analysis for SDE genes in ORF8 TCR-TN mice infected with MHV-68

Using a cut off of 5 RPKM for any genes in either of the conditions and two fold differential expression, we selected 1667 significantly differentially expressed (SDE) genes for network analysis. Network analysis was done using STRING (Search Tool for the Retrieval of Interacting Genes, https://string-db.org/) online tool. To filter out noise from the signals, we used stringent conditions. We obtained a protein-protein interaction (PPI) network that had an enrichment p value of less than 1.0e-16. Network with 498 SDE genes forming Nodes out of 1667 was obtained with STRING using default parameters. The STRING generated a .tsv file as an output that contained node1 (gene of our interest), node 2 (interacting partner). The .tsv file (output from STRING) were opened in excel and analyzed for hub genes. Genes having more than five interacting partners yielded a total of 229 hub genes (Table S5). A gene was considered as a hub gene if it had more than 5 degree of centrality. The degree of centrality $Cd(i) = deg(i) = |N(i)|$, where deg (i) is the degree of node (i), and $|N(i)|$ is the number of neighboring nodes to the node (i) (Pavlopoulos et al., 2011). To further resolve the initially generated STRING network (that had genes which were not interacting), hub gene network was further made by STRING. Finally STRING network was generated to show Lgals genes as hub genes.

Phenotypic analysis of cells using flow cytometry

Spleen samples were extracted from different groups of animals and single cell suspensions were prepared. $1x10^6$ splenocytes were used for surface staining. Prior to the addition of specific antibody, cells were blocked with anti-Fc receptor antibody (CD16/32) for 20 min at 4°C. 1μg/ml of respective fluorochrome-labeled antibodies was added to the cell suspension and incubated for 30 minutes. After three rounds of washing with PBS-2%FCS, cells were analyzed by flow cytometry. To measure the

relative expression of galectin-3 intracellularly and on the surface of activated cells, we performed surface staining as well as intracellular staining. Splenocytes from C57BL/6 mice were activated with anti-CD3 and anti-CD28 for 48 hrs at 37 degree C. After different time intervals cells were stained with anti-galectin 3, anti- CD69 and anti-CD8. For intracellular cytokine staining (ICCS), $1x10^6$ of freshly isolated splenocytes or LN cells were cultured in 96-well round-bottomed plates in the presence of various concentrations of the indicated peptides and brefeldin A (5µg/ml) for 5 hr at 37°C in a humidified CO_2 incubator. At the end of the incubation period, cell surface staining, followed by ICCS was performed using a cytofix/cytoperm kit (BD Bioscience) as per manufacturer's instructions. Stained cells were analyzed flow cytometry using a FACScalibur (BD Bioscience) instrument and data were analyzed using Flowjo software (Tree star, OR). Cells were then analyzed by flow cytometry.

Immunofluorescence staining, confocal microscopy and co-localization analysis

To examine co-localization of galectin-3 with other molecules involved in immunological synapse formation during antigen-specific $CD8^+$ T cell activation, we performed confocal microscopy. SIINFEKL-specific endogenous or OT1 cells were activated by any of the following three approaches; class I MHC tetramer ($H-2K^b$ -SIINFEKL-tetramers-allophycocyanin)-coated coverslips, SIINFEKL-peptide pulsed BMDCs or magnetic beads coated with $H-2K^b$-SIINFEKL monomers. We investigated co-localization of galectin-3 with TCR marked by SIINFEKL peptide-loaded class I MHC tetramer ($H-2K^b$ -SIINFEKL-tetramer-allophycocyanin) and Zap70, a TCR-CD3 ζ-chain associated protein kinase involved in the initial stages of TCR signaling Confocal laser scanning microscope from Olympus (FV10i-LIV / FV10i-DOC) that has a speed of 1.1 fps having 256 x 256 - 1024 × 1024 pixel resolution and FV10i-DOC: NA 0.4 /NA 1.35 objective was used for acquiring images. Fiji software was used for analyzing the images. This software was used to calculate the Pearson's correlation coefficient (PCC) for co-localization of the probes used as described

elsewhere (Dunn et al., 2011). To ascertain whether or not galectin-3 produced by $CD8^+$ T cells act intracellularly or extracellularly to control their activation, we performed neutralization experiments using α-lactose. α-lactose compete with galectins for their binding to carbohydrates and blocks the interaction (Demetriou et al., 2001). SIINFEKL peptide pulsed BMDCs were co-cultured with OT 1 cells in the presence of absence of 100 mM of α-lactose solution and the extent of localization of galectin-3 towards immune synapse was measured. Atleast 35 cells were counted for each group for calculating co-localization percentages for different molecules. Different methods of activation of $CD8^+$ T cells for confocal imaging are described in subsequent sections. To measure whether or not α-lactose indeed works we measured the response of $CD8^+$ T cells in separate experiments. $CD8^+$ T cells were purified from lymph nodes of C57BL/6 mice and incubated with similar dose of α-lactose for 1 hour at 37 C. The cells were then was then washed three times with PBS and stimulated with anti-CD3 (coated) and anti-CD28 (soluble) for 12 hrs at 37 degree C. After activation, the cells were washed and stained with anti-CD69 and CD8 antibodies.

Activation of $CD8^+$ T cells with MHC I tetramers

Class I MHC (H-$2K^b$) SIINFEKL tetramer (allophycocyanin dye-conjugated) was coated on poly-lysine (Sigma Aldrich)-treated coverslips overnight at 4°C. The next day, coverslips were washed with sterile PBS and complete RPMI. SIINFEKL specific Sorted CD8 T cells were added to these coverslips and incubated for different time intervals. Control cells were immobilized on poly-lysine treated coverslips in the absence of Class I MHC (H-$2K^b$) SIINFEKL tetramers. After incubation, cells were washed gently with PBS and were fixed and permeabilized using buffers from eBioscience (Intracellular fixation buffer and intracellular permeabilization buffer). This was followed by blockade with 5% FBS in permeabilization buffer. Cells were stained with mouse anti-galectin 3 (Thermofisher)

and rabbit anti-Zap70 (Cell Signaling Technology) for 2 hrs at room temperature. After washing, cells were stained for 30 minutes with anti-mouse alexa fluor 568 (Thermofisher) and anti-rabbit alexa fluor 488 (Thermofisher). Thereafter cells were washed and mounted with fluoromount (Sigma Aldrich).

Activation of CD8$^+$ T cells with peptide pulsed APCs

APCs were generated from the bone marrow of C57BL/6 mice, using IL-4 and GM-CSF as described (Sehrawat et al., 2013). 1x10^5 APCs were placed on poly-lysine-treated coverslips and pulsed with SIINFEKL peptide (1µg/ml) for 2 hrs at 37°C. 4x10^5 purified OT1 CD8$^+$ T were co-cultured with peptide-pulsed BMDCs for upto an hr. Cells were then stained as described in the previous section and analyzed by confocal microscopy.

***In vivo* activated cells analysis by immunofluorescence microscopy**

5x10^4 or 1x10^5 purified CD8$^+$ T cells from OT1 mice were adoptively transferred into gender-matched C57B/6 mice. Subsequently, mice were infected intranasally with 2x10^5 pfu of MHV68-M2-SIINFEKL virus. At 6 or 55 dpi, mediastinal lymph nodes were collected and a single cell suspension was prepared. Cells were stained with class I MHC tetramer and were sorted by FACS. For some experiments, animals were infected with WSN-SIINFEKL and H-2K^b-SIINFEKL-specific CD8$^+$ T cells were isolated at 6 or 40dpi using magnetic beads coated with SIINFEKL-class I MHC monomers. Briefly, one mg of M-270 streptavidin magnetic Dynabeads (In vitrogen) were incubated with 10 µg of biotinylated H-2K^b monomers, that were generated using UV cleavable peptide for two hours at 4°C with gentle shaking (5 rpm). After incubation, one ml of cold PBS was added gently and the beads were magnetically separated. The washing of monomers coated beads was repeated three times. Subsequently, the photocleavable ligand was exchanged with SIINFEKL peptide by UV exposure at 365nm for 60 min and three washings were performed subsequently to remove of any aggregated proteins. The H-2K^b-SIINFELK coated beads thus prepared were mixed with 10x10^6 lymphocytes obtained from the single cell

suspension of spleens and mediastinal LN of virus infected mice. Beads and cells were incubated at 4°C for 90 min with gentle shaking (5rpm). Three times washing was performed with cold PBS using magnetic separation apparatus. The complex of beads and specific cells were added on polylysine-coated coverslips for 10 minutes at 37°C. Specific cells bound with beads were stained for galectin-3, Zap70 and DAPI for their subsequent analysis by confocal microscopy. The efficiency of sorting was ascertained cytofluorimetrically.

Generation of BMDCs

Bone marrow cells were isolated from the long bones of C57BL/6 mice. Cells were cultured with IL-4 (10ng/ml) and GM-CSF (10ng/ml) for four days in a humidified CO_2 incubator at 37°C. After differentiation cells were analyzed by flow cytometery, using anti-CD11b, CD11c and Class II MHC antibodies. More than 70 percent of cells were differentiated into DCs, as judged by expression of CD11c and activation associated molecules such as CD80, CD86 and Class II MHC.

Proliferation of cells

MACS-purified $CD8^+$ T cells from galectin-3 KO and WT animals were labeled with CFSE using protocols described elsewhere (Sehrawat et al., 2012). Labeled cells were stimulated with plate-bound anti-CD3 (1µg/ml) and soluble anti-CD28 (1µg/ml) for varying times. Cells were then collected, washed and stained on ice with the indicated fluorochrome-labeled antibodies. After multiple rounds of washing with FACS buffer, cells were analyzed by flow cytometry. Culture supernatants were collected to measure secreted cytokines. To assess the influence of extracellular galectin-3 on the proliferation of $CD8^+$ T cells, $10x10^6$ OT1 cells were labeled with 2.5µM CFSE and $2x10^5$ of labeled cells were stimulated with plate bound anti-CD3 (1ug/ml) and soluble anti-CD28 (1µg/ml) antibodies in the presence or absence of anti-galectin-3 neutralizing antibody (10µg/ml). Cells were incubated at 37°C and CFSE dilution was measured at different time points.

ELISA for cytokine measurement

Culture supernatants were collected from stimulated cells, supplemented with a protease inhibitor cocktail (Roche Diagnostics) and stored at -20°C until use. The levels of IL-2 in the culture supernatants were measured by sandwich ELISA using the OptEIA kit for mouse IL-2 (BD Bioscience).

Extraction of lung tissue for virus titration

Lung tissues were collected from WT and Gal-3 KO mice infected intranasally with $5x10^5$ pfu of MHV-68 and euthanized by CO_2 asphyxiation six days post-infection. Blood vessels just above the liver were cut and mice were perfused with 10-15 ml of sterile PBS by inserting a 25G needle into the right ventricle of the exposed heart. Lung tissues were weighed and frozen until use. Prior to titration of virus on 3T12-3 cells, lung tissues were thawed and homogenized with 500µl of DMEM without serum.

Statistical analysis

Student's “t” test and ANOVA tests were applied for statistical analysis to compare responses between groups, as indicated in the figure legends. The results are presented as mean ± SD. The p values are shown in the figures or figure legends and are represented as *$p \le 0.05$, **$p \le 0.01$, or ***$p \le 0.001$.

Supplementary references

Altman, J.D., Moss, P.A., Goulder, P.J., Barouch, D.H., McHeyzer-Williams, M.G., Bell, J.I., McMichael, A.J., and Davis, M.M. (1996). Phenotypic analysis of antigen-specific T lymphocytes. Science *274*, 94–96.

Bakker, A.H., Hoppes, R., Linnemann, C., Toebes, M., Rodenko, B., Berkers, C.R., Hadrup, S.R., van Esch, W.J.E., Heemskerk, M.H.M., Ovaa, H., et al. (2008). Conditional MHC class I ligands and peptide exchange technology for the human MHC gene products HLA-A1, -A3, -A11, and -B7. Proc. Natl. Acad. Sci. U. S. A. *105*, 3825–3830.

Demetriou, M., Granovsky, M., Quaggin, S., and Dennis, J.W. (2001). Negative regulation of T-cell activation and autoimmunity by Mgat5 N-glycosylation. Nature *409*, 733–739.

Dunn, K.W., Kamocka, M.M., and McDonald, J.H. (2011). A practical guide to evaluating colocalization in biological microscopy. AJP Cell Physiol. *300*, C723–C742.

Pavlopoulos, G.A., Secrier, M., Moschopoulos, C.N., Soldatos, T.G., Kossida, S., Aerts, J., Schneider, R., and Bagos, P.G. (2011). Using graph theory to analyze biological networks. BioData Min. *4*, 10.

Sehrawat, S., Kirak, O., Koenig, P.-A., Isaacson, M.K., Marques, S., Bozkurt, G., Simas, J.P., Jaenisch, R., and Ploegh, H.L. (2012). CD8(+) T cells from mice transnuclear for a TCR that recognizes a single H-2K(b)-restricted MHV68 epitope derived from gB-ORF8 help control infection. Cell Rep. *1*, 461–471.

Sehrawat, S., Koenig, P.-A., Kirak, O., Schlieker, C., Fankhauser, M., and Ploegh, H.L. (2013). A catalytically inactive mutant of the deubiquitylase YOD-1 enhances antigen cross-presentation. Blood *121*, 1145–1156.
